# Stearoyl‐CoA Desaturase‐1 Drives Tumor Growth by Interacting With Histone Deacetylase‐2 and Deacetylating Nucleophosmin‐1

**DOI:** 10.1002/mco2.70809

**Published:** 2026-06-11

**Authors:** Coline Wery, Laetitia Montero‐Ruiz, Eric Bonneil, Mohammad Farran, Robin Jehay, Quentin Herrara Garfia, Gregory Fettweis, Silvia Blacher, Charles Pottier, Gael Cobraiville, Yasmine Boumahd, Olivier Peulen, Agnès Noël, Franck Dequiedt, Marianne Fillet, Nor Eddine Sounni

**Affiliations:** ^1^ Cancer Metabolism and Tumor Microenvironment Lab GIGA‐Cancer GIGA Institute University of Liège Liège Belgium; ^2^ Laboratory of Tumor and Development Biology GIGA Institute University of Liège Liège Belgium; ^3^ Institute for Research in Immunology and Cancer (IRIC) University of Montréal Montréal Canada; ^4^ Laboratory of Gene Expression and Cancer GIGA Institute University of Liège Liège Belgium; ^5^ Laboratory for the Analysis of Medicines CIRM Institute University of Liège Liège Belgium; ^6^ Metastasis Research Laboratory GIGA‐Cancer GIGA Institute University of Liège Liège Belgium

**Keywords:** cancer drug resistance, HDACs, lipid droplets, nonhistone protein deacetylation, NPM1, SCD1

## Abstract

The adaptation of lipid metabolism in cancer cells, driven by changes in the tumor microenvironment, presents major challenges for cancer therapy. Here, we addressed the problem of altered lipid metabolism and its role in cancer progression and therapeutic resistance. We demonstrate that hypoxia upregulates the key desaturase, stearoyl‐CoA desaturase‐1 (SCD1), and the lipid droplet (LD) protein PLIN2, thus promoting lipid metabolic adaptation, cell proliferation, migration, and tumor growth. We found that SCD1 and PLIN2 are essential and interdependent for LD formation. PLIN2 supports cell survival under hypoxic and metabolic stress, whereas SCD1 sustains cancer cell proliferation upon reoxygenation. In addition, we found that SCD1 expression in cancer cells affects nonhistone protein deacetylation, whereas PLIN2 expression enhances protein acetylation. Among these proteins, nucleophosmin(NPM1), a tumor suppressor and regulator of p53, was destabilized through SCD1‐dependent deacetylation. In addition, SCD1 interacts with NPM1, influences its cellular localization, and recruits histone deacetylase‐2 (HDAC2) to the complex. Notably, we observed that knockdown of SCD1 in vitro or its pharmacological inhibition in vivo enhances cancer cell sensitivity to HDAC inhibitors. Our findings underscore the role of SCD1 in reshaping the cellular acetylome and suggest that targeting SCD1 could sensitize cancer cells to HDAC inhibitors, highlighting a promising therapeutic strategy.

## Graphical abstract

Hypoxia induces SCD1 and PLIN2 to drive lipid droplet‐dependent metabolic adaptation in cancer cells. PLIN2 supports survival under stress, whereas SCD1 promotes proliferation upon reoxygenation and remodels the acetylome via HDAC2‐mediated NPM1 deacetylation and destabilization. Targeting SCD1 enhances sensitivity to HDAC inhibitors, suggesting a combinatorial therapeutic strategy.

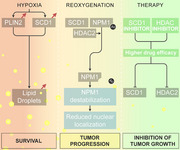

## 1 Introduction

The dynamic metabolic plasticity of cancer and stromal cells poses a significant barrier to effective cancer therapy [1, 2, 3, 4]. Treatments targeting proliferating tumor or stromal cells often encounter drug resistance. Understanding the mechanisms underlying metabolic plasticity in tumors and changes in the tumor microenvironment is essential for improving drug efficacy and response. One notable adaptation is hypoxia‐induced metabolic switching within tumors [5, 6, 7], where cells shift from oxidative phosphorylation to glycolysis, a process known as the Pasteur effect [8]. While the regulation of glycolysis by hypoxia has been extensively studied [9, 10, 11, 12], the role of hypoxia in the control of fatty acid (FA) metabolism in cancer progression and the therapeutic response has received relatively little attention [13, 14].

Lipids, particularly FAs, are crucial building blocks for cellular membrane structure and signaling [15, 16, 17]. While they are central to energy storage and cellular function, FAs can become toxic if not properly utilized or stored in lipid droplets (LDs), potentially triggering cell death [18, 19]. For example, prolonged exposure of cancer cells to palmitic acid can induce apoptosis through an ER stress‐dependent pathway [20]. The alteration of FA metabolism is a hallmark of cancer progression and metastasis. Elevated FA levels, whether originating from dietary sources or changes within the tumor microenvironment, are associated with poor clinical outcomes in various cancers [21, 22, 23]. Recently, LDs have garnered interest because of their regulation during cellular and environmental stress [18, 24, 25, 26]. As essential organelles, LDs play a central role in lipid and energy homeostasis, buffering cells against toxic lipid accumulation and preventing lipotoxicity and oxidative stress [27, 28]. We previously reported that the abundance of LDs increases under therapy‐induced hypoxia, helping protect cancer cells from oxidative stress and ferroptosis [25, 29].

Cancer cell proliferation requires a supply of saturated fatty acids (SFAs) and monounsaturated fatty acids (MUFAs) for energy storage, signaling, and membrane biogenesis [13, 17, 30]. This demand is met by key enzymes involved in de novo FA synthesis, including fatty acid synthase (FASN), ATP citrate lyase (ACLY), and stearoyl‐CoA desaturase‐1 (SCD1), all of which are elevated in proliferating cancer cells and are considered potential drug targets [30, 31, 32]. SCD1 is crucial for proliferation across various cancer types [20, 33, 34, 35], and its expression is associated with ferroptosis resistance and tumor recurrence [25, 36]. However, the mechanisms through which SCD1 influences cancer cell phenotypic plasticity remain poorly understood.

In this study, we show that the expression of SCD1 and the LD‐associated protein PLIN2 is upregulated by hypoxia in cancer cells. Knockdown of PLIN2 or SCD1 resulted in fewer LDs in hypoxic cells, reduced cell migration and proliferation in vitro, and inhibited tumor growth in vivo. Posttranslational modification (PTM) analysis of acetylated proteins in cells depleted of SCD1 and PLIN2 revealed distinct patterns of nonhistone protein acetylation and deacetylation. Specifically, PLIN2 increased nonhistone protein acetylation, whereas SCD1 decreased it. We found that SCD1 deacetylates NPM1, a tumor suppressor protein [37, 38, 39, 40], and consequently reduces its stability. Coimmunoprecipitation (CoIP) and protein complementation assays using Gaussia luciferase (gPCA) demonstrated interactions between SCD1, NPM1, and HDAC2 in molecular complexes. Interestingly, knockdown of SCD1 or its pharmacological inhibition enhances cancer cell sensitivity to HDAC inhibitors (HDACi) both in vitro and in vivo.

## 2 Results

### 2.1 Hypoxia Upregulates the Expression of SCD1 and PLIN2 in Cancer Cells

**FIGURE 1:**
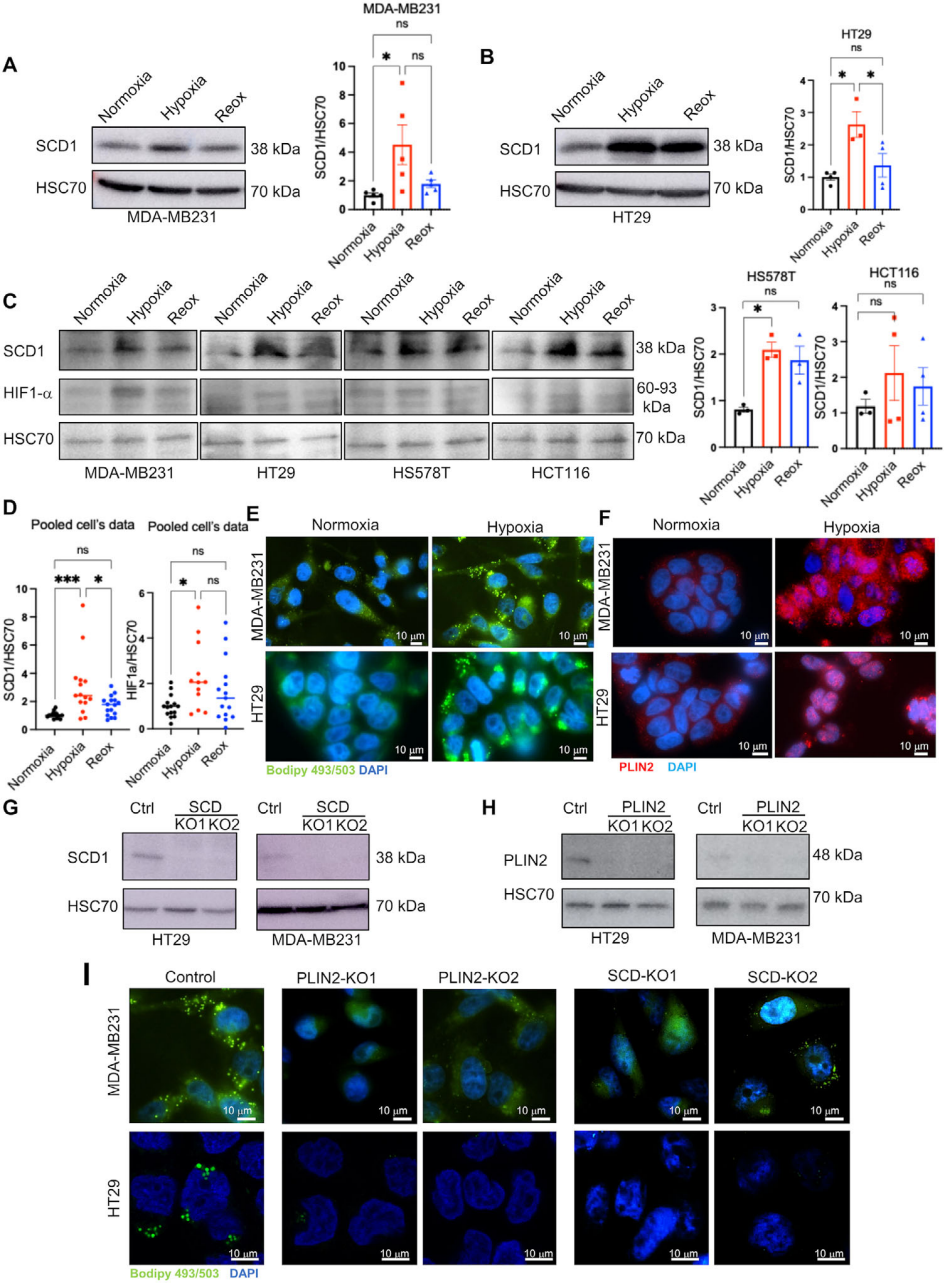
Hypoxia regulates the expression of proteins involved in lipid desaturation and storage in cancer cells. (A and B) Western blot analysis of SCD1 expression in MDA‐MB231 in (A) and HT29 cancer cells in (B) incubated under normoxic or hypoxic conditions and after reoxygenation (Reox). The graphs show the results of the densitometry quantification of the Western blot results. HSC70 served as a loading control. (C) Western blot analysis of SCD1 and HIF1alpha expression in multiple cell lines incubated under normoxic or hypoxic conditions and after reoxygenation (Reox). The graphs show the results of the densitometry quantification of the Western blot results. HSC70 served as a loading control. (D) Pooled densitometric quantification of SCD1 (left) and HIF1α (right) expression by Western blot, normalized to HSC70, under normoxia, hypoxia, and after reoxygenation across all cell lines shown in (A–C). (E) Detection of lipid droplets (LDs) after Bodipy 493/503 staining of MDA‐MB231 and HT29 cancer cells incubated under normoxic or hypoxic conditions. (F) LDs were detected by immunofluorescence staining of PLIN2 in MDA‐MB231 and HT29 cancer cells incubated under normoxic or hypoxic conditions. Scale bars = 10 µm. (G) Western blot analysis of SCD1 expression in control and SCD knockout (SCD‐KO) cells. (H) Western blot analysis of PLIN2 expression in control and PLIN2 knockout (PLIN2‐KO) cells. HSC70 served as a loading control. (I) Detection of LDs in control and PLIN2‐KO cells after Bodipy 493/503 staining. Both the Western blot and immunofluorescence (IF) experiments were independently repeated three times (*n* = 3). ^*^ *p* < 0.05 and ^***^ *p* < 0.001.

To explore the effects of hypoxia on the expression of lipid metabolism proteins, MDA‐MB231 and HT29 cells were incubated under hypoxia for 24 h, after which the expression of key proteins involved in lipid desaturation and storage was analyzed. SCD1 expression increased under hypoxic conditions but slightly decreased after reoxygenation, indicating a cellular response to hypoxic stress (Figure 1A,B). Similar results were observed in additional cell lines, including HS578T and HCT116 cells, whose expression increased under hypoxia but modestly decreased after reoxygenation (Figure 1C). Successful induction of hypoxia was confirmed by the increased expression of HIF1α in all the tested cell lines. No change in SCD1 mRNA levels was detected in MDA‐MB‐231, HT29, HS578T, or HCT116 cells incubated under hypoxia (Figure S1A). However, reoxygenation led to an increase in SCD1 transcript levels, with significant changes observed in HT29 and HCT116 cells. Consistent with the mRNA profile of SCD1, the expression of the transcription factors SREBP1 and SREBP2 did not significantly change under hypoxia in any of the four cell lines (Figure S1B,C). Bioinformatic analysis of human tumor datasets (pan‐cancer, breast, and intestinal cancer datasets) revealed a positive correlation between HIF1α and SCD1 expression (Figure S1D). While the transcriptional regulation of SCD1 may play a role in human tumors, our in vitro data indicate that hypoxia primarily regulates SCD1 and HIF1α at the protein level in all four tested cell lines (Figure 1A–C), as well as in pooled analyses across these lines (Figure 1D). Strong association between HIF1α expression and lipid metabolism pathways were consistently observed across human cancer datasets, and Gene Ontology (GO) pathway analysis of ARCHS4 RNA sequencing data revealed that an LD signature was significantly correlated with HIF1A expression (Figure S1D,E). These findings prompted us to examine LD abundance in our cell models.

Bodipy staining and immunofluorescence for PLIN2 confirmed the increase in LD formation under hypoxia (Figure 1E,F), highlighting LDs as a cellular adaptation to hypoxic stress. CRISPR‐Cas9‐mediated knockout (KO) experiments demonstrated that SCD1 and PLIN2 are required for LD formation under hypoxia, as KO cells did not form LDs under these conditions (Figure 1G–I and Figure S1F). Hypoxic conditions increased PLIN2 expression in both cell lines. However, this increase was inhibited when the cells were treated with an SCD1 inhibitor (SCD1i) (Figure S2A). Similarly, SCD1 expression was reduced in PLIN2‐KO cells under hypoxia (Figure S2B), suggesting the interdependence of SCD1 and PLIN2. This connection was further validated by the inhibition of PLIN2+ LDs when cancer cells were incubated under hypoxia and treated with SCD1i (Figure S2C). Furthermore, SCD1 and PLIN2 were coimmunoprecipitated (Figure S2D), which was confirmed by reverse IP (Figure S2E), suggesting that these two proteins are associated with each other and support their functional connection during cellular adaptation to hypoxic stress.

### 2.2 The Inhibition of PLIN2 or SCD1 Expression Reduces Cancer Cell Proliferation Both In Vitro and In Vivo

**FIGURE 2:**
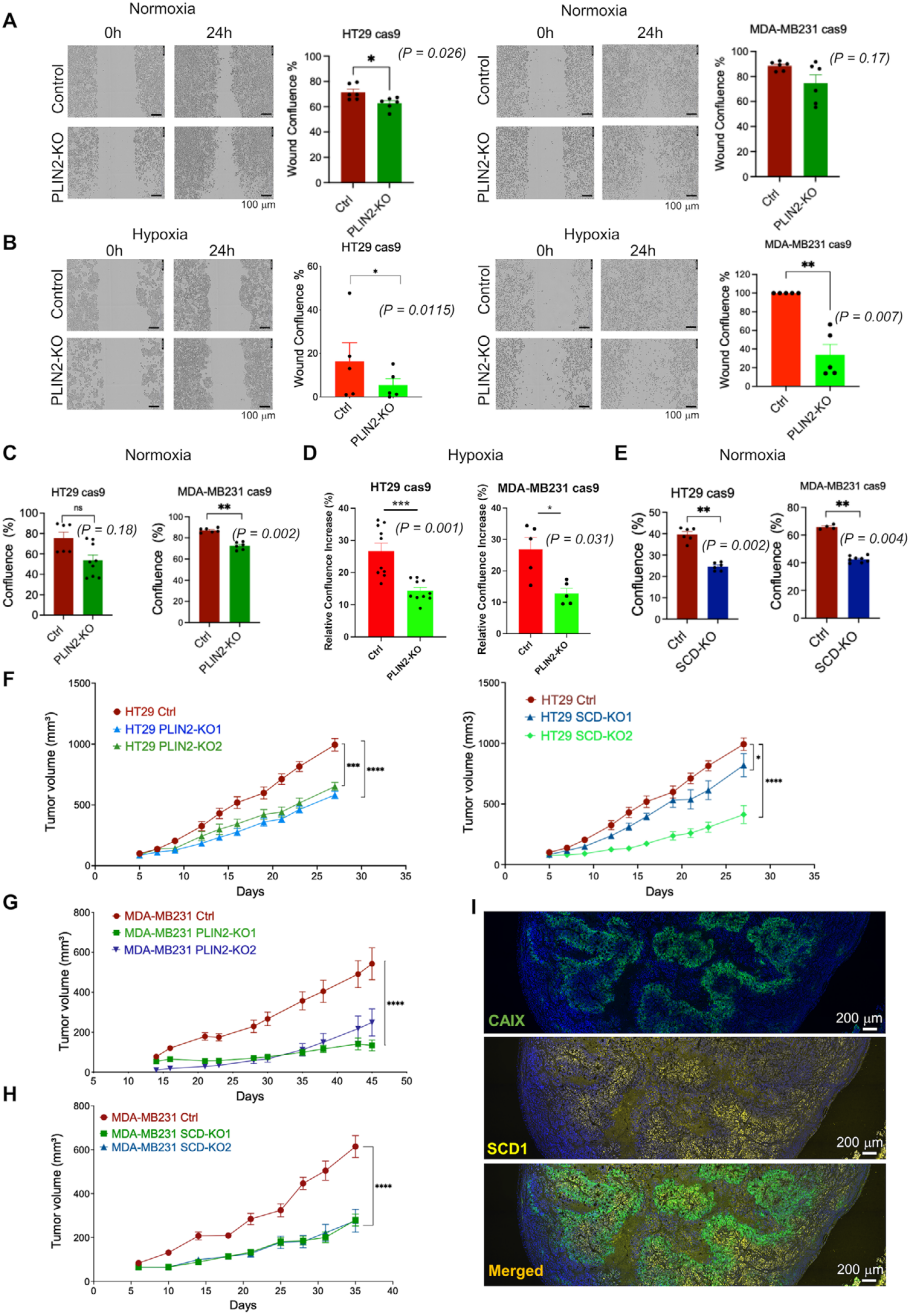
PLIN2 and SCD1 inhibition reduces cancer cell proliferation both in vitro and in vivo. (A) Assessment of the migration of HT29 (left) and MDA‐MB231 (right) control cells (expressing the Cas9 enzyme and the non‐targeted gRNA control) and PLIN2‐KO cancer cells incubated under normoxic conditions for 24 h. (B) Assessment of the migration of HT29 (left) and MDA‐MB231 (right) control and PLIN2‐KO cancer cells under hypoxic conditions for 24 h. Scale bars = 100 µm. (C) Proliferation assessment of HT29 (left) and MDA‐MB231 (right) control and PLIN2‐KO cancer cells incubated under normoxic conditions for 24 h. (D) Assessment of the proliferation of HT29 (left) and MDA‐MB231 (right) control and PLIN2‐KO cancer cells incubated under hypoxic conditions for 24 h. (E) Proliferation assessment of the HT29 (left) and MDA‐MB231 (right) control and SCD‐KO cells incubated under normoxic conditions. (F) Tumor growth of HT29 control (Ctrl), PLIN2‐KO1 and PLIN2‐KO2 cells (left graph), and SCD‐KO cells (SCD‐KO1 and SCD‐KO2) (right graph) injected into RAG2^−/−^ mice (*n* = 6); the HT29 control cells on the left and on the right are identical. (G and H) Growth of tumors formed from MDA‐MB231 control (Ctrl), PLIN2‐KO (PLIN2‐KO1 and PLIN2‐KO2) cells (G), and SCD‐KO (SCD‐KO1 and SCD‐KO2) cells in (H), injected into nude mice (*n* = 6). (I) Immunofluorescence staining of CAIX (green) and SCD1 (yellow) in MDA‐MB231 control tumors, showing whole sections and the adjacent localization of CAIX and SCD1. Scale bars = 200 µm. ^*^ *p* < 0.05, ^**^ *p* < 0.01, and ^***^ *p* < 0.001.

We investigated the effects of PLIN2 and SCD1 depletion on cancer cell migration, proliferation, and tumor growth under different oxygen conditions. Under normoxia, PLIN2 depletion slightly reduced HT29 cell migration but did not significantly affect MDA‐MB231 cells (Figure 2A). In contrast, the migration of both cell types depleted of PLIN2 was notably inhibited under hypoxia (Figure 2B). Cell proliferation was next monitored over a 24‐h period using *Incucyte*. PLIN2 depletion under hypoxia resulted in a pronounced inhibition of cell proliferation in both cell lines, whereas under normoxia, a slight reduction in proliferation was observed only in MDA‐MB231 cells (Figure 2C,D). SCD1 depletion under normoxia significantly inhibited proliferation in both cell lines (Figure 2E). Given the oxygen required for its enzymatic activity, we did not test the effects of hypoxic conditions on SCD1‐depleted cells in vitro. In vivo, both PLIN2 and SCD1 depletion led to reduced tumor growth in the HT29 and MDA‐MB231 xenografts, supporting their protumorigenic effects (Figure 2F–H). Immunohistochemical analyses of tumor sections revealed a reduction in Ki67‐positive cells in PLIN2‐depleted tumors for both cell lines, indicating decreased proliferation (Figure S3A). No significant change was observed in the number of Ki67‐positive cells in SCD1‐depleted tumors. Analysis of the expression of an apoptotic marker (caspase‐3) revealed an increase in the number of apoptotic cells only in PLIN2‐depleted MDA‐MB231 tumors but not in those in HT29 tumors, and no difference in cells depleted of SCD1 (Figure S3B). To visualize hypoxic regions within the tumors and assess SCD1 expression in the xenografts, we performed immunofluorescence staining for CAIX and SCD1 (Figure 2I). SCD1 expression was consistently detected adjacent to CAIX‐positive hypoxic areas, and this pattern was observed in all the sections from both the HT29 and MDA‐MB‐231 xenografts. In SCD1‐KO xenografts, SCD1 staining was markedly reduced within the tumor compartment, particularly in CAIX‐positive hypoxic regions (Figure S3C), with the remaining signal likely attributable to mouse stromal cells. These observations further support a strong association between hypoxia and SCD1 expression, highlighting a potential protumorigenic role for SCD1 in hypoxic and metabolically stressed tumor microenvironments.

### 2.3 PLIN2 Inhibition Affects Fatty Acid Oxidation in Cancer Cells

**FIGURE 3:**
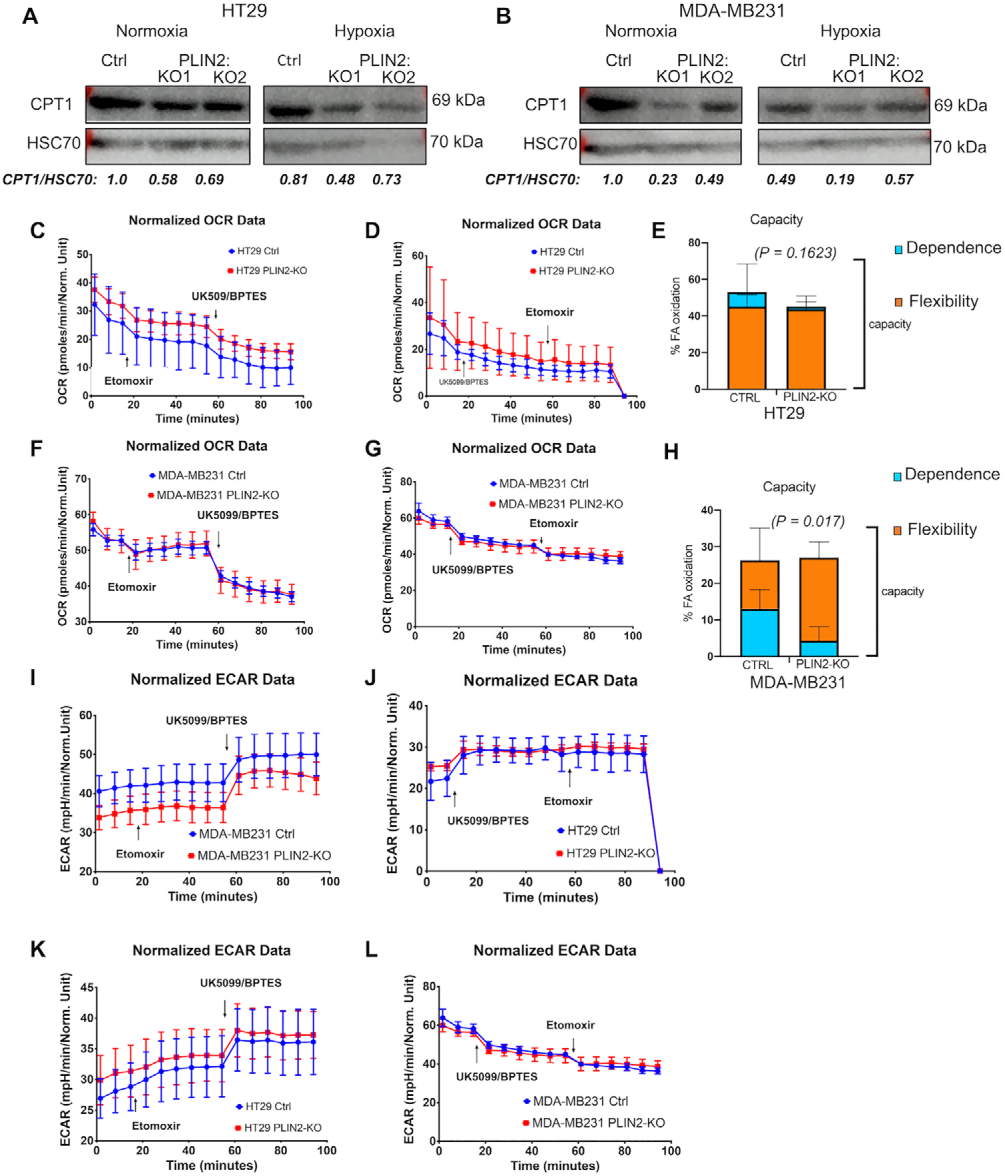
Decreased dependency on fatty acid oxidation (FAO) in PLIN2‐depleted cells. (A and B) Western blot of CPT1 in control and PLIN2‐KO HT29 cells (A) and MDA‐MB231 cells (B) incubated under normoxic and hypoxic conditions for 24 h. HSC70 served as a loading control. The results of the densitometry analysis of CPT1 were normalized to HSC70, and the values are shown below the blots. Control and PLIN2‐KO HT29 and MDA‐MB231 cells were preincubated under hypoxia for 24 h, and their metabolic activities were assessed. (C and D) Oxygen consumption rate (OCR) normalized capacity after incubation with the fatty acid oxidation inhibitor (etomoxir) or a combination of pyruvate and the glutamine inhibitors (UK5099/BPTES). (E) Fatty acid oxidation percentage representing capacity (Dependence + Flexibility), dependence (Blue) and flexibility (Orange) in control and PLIN2‐KO HT29 cells. (F and G) Normalized OCR data from dependence (F) or capacity (G) measurements in control and PLIN2‐KO MDA‐MB231 cells. (H) Fatty acid oxidation percentage representing capacity (Dependence + Flexibility), dependence (Blue) and flexibility (Orange) in MDA‐MB231 cells. (I and J) Normalized extracellular acidification rate (ECAR) data from dependence (I) and capacity (J) measurements in control and PLIN2‐KO HT29 cells. (K and L) Normalized ECAR data from dependence (K) and capacity (L) measurements in control and PLIN2‐KO MDA‐MB231 cells. WB analyses were conducted in two independent repeats (*n* = 2), while OCR and ECAR measurements were obtained from a single experiment performed in technical triplicates.

Assuming that PLIN2 is essential for LD formation, we assessed fatty acid oxidation (FAO) dependency in PLIN2‐depleted cancer cells. Western blot analysis revealed that the expression of CPT1, the rate‐limiting enzyme in FAO, was reduced in PLIN2‐depleted cells under normoxia but was not affected by hypoxic conditions (Figure 3A,B). The cells were subsequently incubated under hypoxia for 24 h. FAO dependency and flexibility were evaluated using a Mito Fuel Flex Test Kit. Cancer cells were sequentially incubated with the pathway inhibitors etomoxir (CPT1), UK5099 (pyruvate), and BPTES (glutaminase) in a Seahorse XF Analyzer, after which the oxygen consumption rate (OCR) and extracellular acidification rate (ECAR) were determined. Compared with control cells, PLIN2‐depleted HT29 and MDA‐MB231 cells were able to compensate for FAO inhibition (etomoxir) by using alternative fuel sources and exhibited decreased dependency on FAO (Figure 3C–H). Moreover, PLIN2 depletion did not affect the metabolic flexibility of either cell lines treated with etomoxir or with inhibitors of pyruvate or glutamine. Treatment with etomoxir and UK5099/BPTES inhibitors led to an increase in the ECAR in both cell lines (Figure 3I–L). These data may reflect enhanced glucose consumption as a compensatory response, reflecting metabolic flexibility in response to blocked FAO and glutamine utilization.

### 2.4 The Inhibition of PLIN2 or SCD1 Affects Lipid Synthesis and the Acetylome of Cancer Cells

**FIGURE 4:**
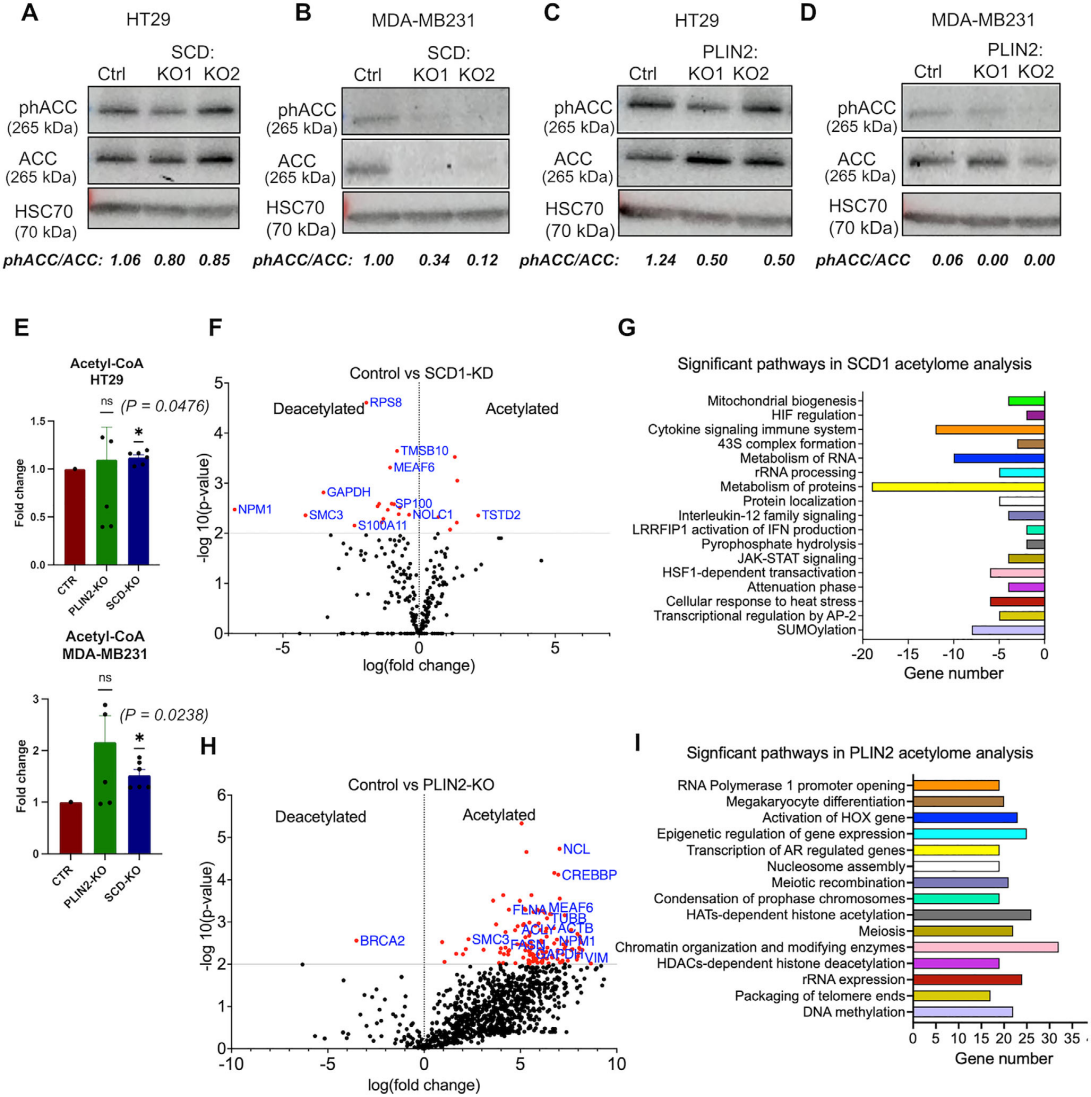
Inhibition of PLIN2 or SCD1 affects lipid synthesis in cancer cells. (A and B) Western blot analysis of phACC and ACC expression in control and SCD‐KO HT29 cells (A) and in MDA‐MB231 cells (B). (C and D) Western blot analysis of phACC and ACC in control and PLIN2‐KO HT29 cells (C) and in MDA‐MB231 cells (D). HSC70 served as a loading control. The densitometry of phACC/total ACC ratios were normalized to the control, and the values are shown below the blots. (E) Mass spectrometry quantification of the acetyl‐CoA pool, presented as fold changes over control cells, in SCD‐KO and PLIN2‐KO HT29 and MDA‐MB231 cells. (F) Volcano plot of the proteins in acetylome scan analysis of control versus SCD1‐depleted MDA‐MB231 (SCD1‐KD) cells and according to the average ratio of three technical replicates and *p* values. (G) Graphical representation of pathways involving proteins that are differentially deacetylated by SCD1. (H) Volcano plot of the proteins identified by acetylome scan analysis in control versus PLIN2‐depleted MDA‐MB231 cells according to the average ratio of three technical replicates and *p* values. (I) Graphical representation of pathways involving proteins that are differentially acetylated by PLIN2. Each experiment was repeated twice (*n* = 2), except for the acetylome analysis, which was performed once in biological triplicate.

We investigated the effects of PLIN2 and SCD1 depletion on de novo FA synthesis and protein PTMs in cancer cells. Western blot analysis of FASN, ACC, and pACC revealed no significant changes in FASN levels (Figure S4A–D) but revealed reduced ACC and pACC levels in PLIN2‐ and SCD1‐depleted MDA‐MB231 cells, suggesting a decrease in FA synthesis (Figure 4A–D). However, no striking effect on the acetyl‐CoA pool was detected in MDA‐MB231 and HT29 cells depleted of SCD1 or PLIN2, whereas a slight fold change increase was detected in SCD‐KO MDA‐MB231 cells (Figure 4E). We then performed a posttranslational acetylome scan analysis by proteomics in cells depleted of SCD1 or PLIN2, compared with control cells. Proteomic analysis revealed an increase in the acetylation of nonhistone proteins, including NPM1, SMC3, GAPDH, and VIM, in SCD1‐depleted MDA‐MB231 cells, but these proteins were deacetylated in control cells (Figure 4F and Figure S5A). Pathway analysis indicated that SCD1 depletion affected mainly protein metabolism and cytokine signaling pathways (Figure 4G). In contrast, PLIN2 depletion was linked to decreased acetylation of nonhistone proteins (Figure 4H and Figure S5B), affecting pathways related to epigenetic regulation, chromatin modification, the cell cycle, RNA metabolism, and SUMOylation (Figure 4I and Figure S5C). Notably, among the most prominently modified proteins, NPM1 exhibited significant deacetylation (100‐fold) by SCD1 (Figure S5A), highlighting its role in the cell cycle and stress response.

### 2.5 SCD1 Interacts With NPM1 and Increases Its Stabilization

**FIGURE 5:**
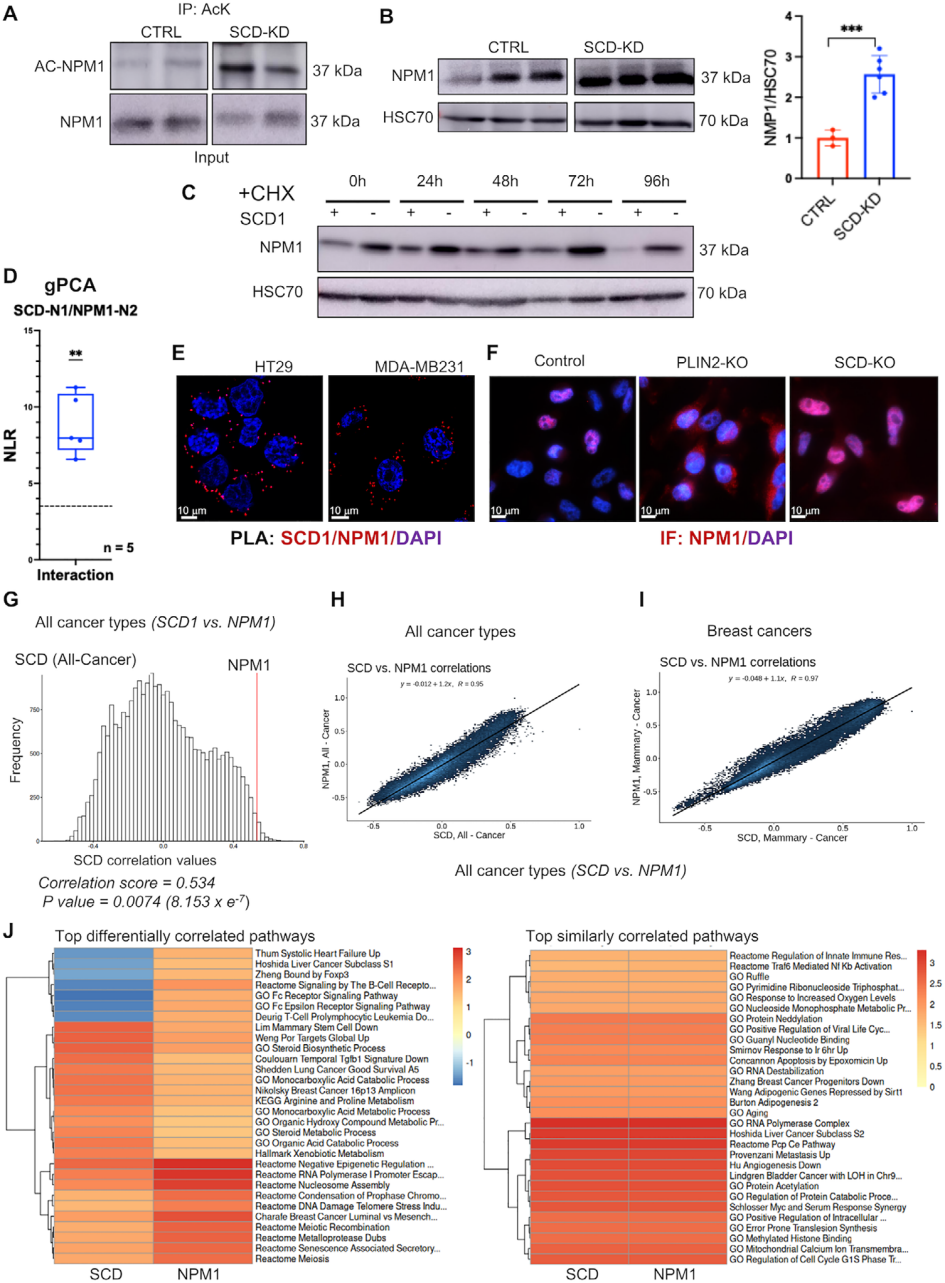
SCD interacts with NMP1 and increases its stabilization. (A) Coimmunoprecipitation of acetylated‐lysines (AcK) and detection of NPM1 by Western blot in control and SCD1‐depleted cells. (B) Western blot analysis of NPM1 in cell lysates of control and SCD1‐depleted cells, with densitometric quantification. (C) Stability assay for NPM1 by Western blot at different time points after treatment with cycloheximide (CHX) (100 µM) in control and SCD1‐depleted cells. HSC70 served as a loading control. (D) The interaction between SCD1 and NPM1 was assessed using Gaussia protein‐fragment complementation assays (gPCA). The normalized luminescence ratio (NLR) for the SCD1–NPM1 interaction is shown. The experiments were performed with *n* = 5 independent biological replicates. The *p* value corresponds to *p <* 0.01. (E) Proximity ligation assay images of HT29 and MDA‐MB231 cells. Red dots represent the proximity between NPM1 and SCD1. (F) Immunofluorescence staining of NPM1 in control, PLIN2‐KO and SCD‐KO MDA‐MB231 cells. NPM1 is shown in red, and DAPI (blue) indicates the nucleus. Scale bars = 10 µm. (G) Correlation analysis of *SCD1* gene (SCD) expression with that of other genes revealed a correlation score of 0.534 with that of NPM1 in all cancer types (*p* = 0.007435). (H and I) Scatter plot showing the relationships between genome‐wide correlations of coexpression for *SCD1* (SCD) and *NPM1* in all cancer types (H) and in breast cancer types (I). Displayed *R* values were determined by Pearson correlation. (J) Top differentially (left) and similarly (right) correlated pathways between *SCD1* (SCD) and *NPM1* target genes in all cancer types. All experiments, except where indicated, were reproduced at least twice. The correlation analysis used ARCHS4 v8 (Feb 2020) with 238,522 human samples (Illumina HiSeq/NextSeq), each linked to GEO entries with tissue annotations and metadata.

The link between SCD1 and NPM1 deacetylation of NPM1 was confirmed by immunoprecipitation (IP) with acetyl‐lysine antibodies (AcK) followed by Western blot analysis for NPM1. Increased levels of acetylated NPM1 were detected in SCD1‐depleted cells (SCD1‐KD) than in control cells (Figure 5A). In addition, analysis of total NPM1 levels in cell lysates (Figure 5B) revealed that NPM1 was more abundant in SCD1‐KD cells than in control cells, suggesting that acetylation may reduce the stability of NPM1. To address this issue, MDA‐MB231 control and SCD‐KO cells (Figure 5C), as well as HT29 control and SCD‐KO cells, were treated with cycloheximide, a protein synthesis inhibitor (Figure S6A). After 96 h, NPM1 expression was significantly reduced in control cells, whereas the level of NPM1 remained stable in SCD1‐depleted cells following cycloheximide treatment. Furthermore, IP of SCD1 with NPM1 (Figure S6B) confirmed their association with molecular complexes across multiple cell lines, which was further corroborated by reciprocal IP in MDA‐MB231, HT29, HS578T, and HCT116 cells (Figure S6C). To further validate the interaction between SCD1 and NPM1, we performed Gaussia protein‐fragment complementation assays (gPCA) [41, 42] (Figure 5D). The normalized luminescence ratio (NLR) for the SCD1–NPM1 pair was 8, demonstrating a close physical interaction between the two proteins in HEK293T cells. The interaction between SCD1 and NPM1 was further validated by a proximity ligation assay (PLA), which revealed that SCD1 and NPM1 were colocalized in the cytoplasm of MDA‐MB231 and HT29 cells (Figure 5E). Moreover, immunofluorescence (IF) staining revealed increased nuclear localization of highly acetylated NPM1 in SCD‐KO cells compared with that in wild‐type (WT) control cells. Conversely, in PLIN2‐KO cells, compared with control cells, deacetylated NPM1 was less abundant in the nucleus. These results suggest that SCD1 influences the cellular localization of NPM1 (Figure 5F). Overall, our findings demonstrate that the stability and localization of the chaperone protein NPM1 are regulated by SCD1‐dependent deacetylation. In addition, a high correlation score of 0.534 between the *SCD* and *NPM1* genes (*p* = 0.007435) was particularly evident across multiple cancer types, including breast cancer (Figure 5G–I). As a control for the correlation analyses, for instance, no correlation was found between the *SCD* gene and the *VEGFA* gene in human cancers (Figure S6D). Moreover, the expression of the *SCD* and *NPM1* genes revealed several similar common target genes and pathways (Figure 5J and Figure S6E–G), suggesting that SCD1 and NPM1 function together in human cancers.

### 2.6 SCD1 Interacts With HDAC2

**FIGURE 6:**
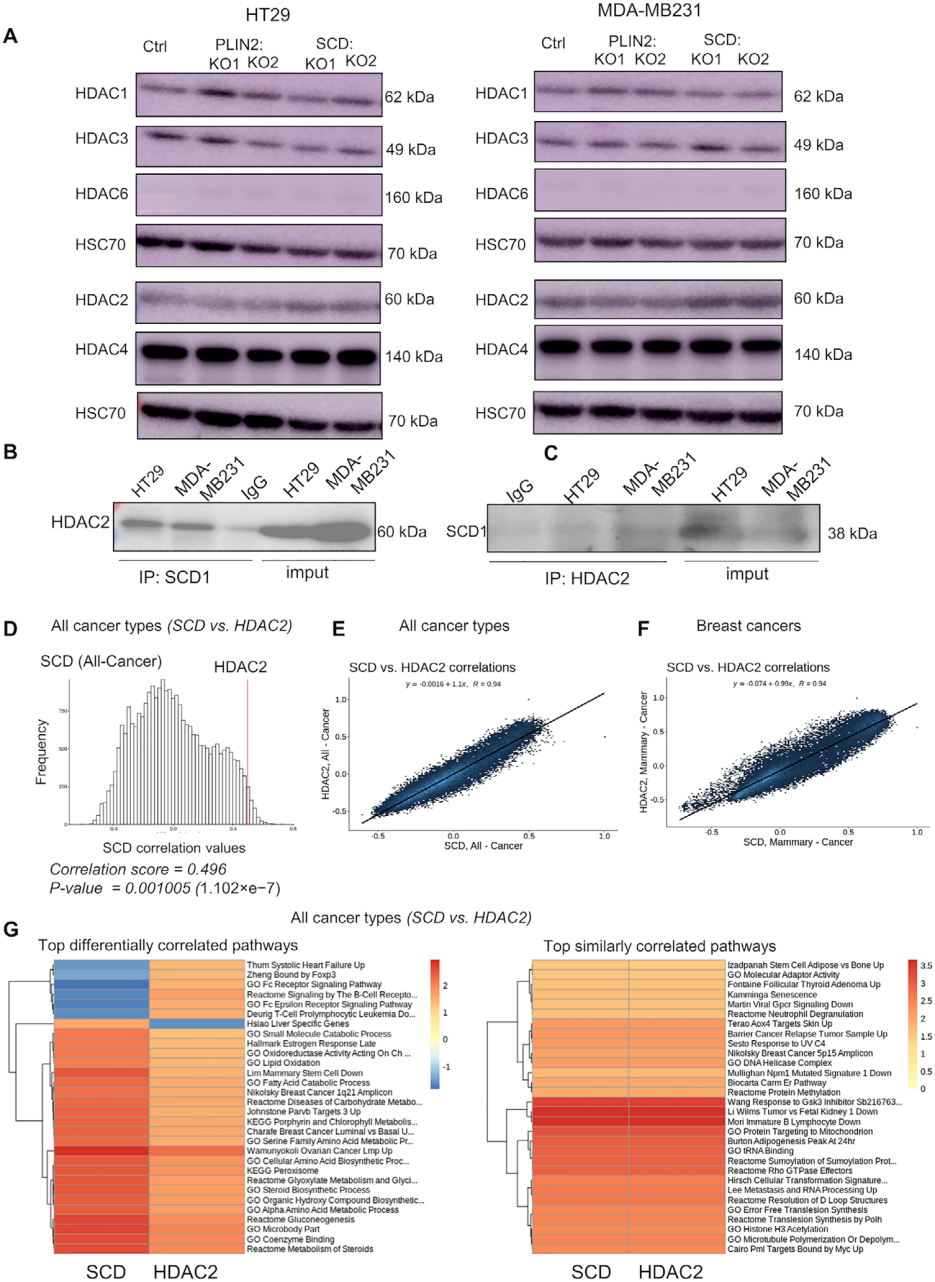
SCD1 interacts with HDAC2. (A) Western blot analysis of HDAC1, 2, 3, 4 and 6 protein expression in control, PLIN2‐KO and SCD‐KO HT29 and MDA‐MB231 cells. HSC70 served as a loading control. (B) Immunoprecipitation (IP) of SCD1 and detection of HDAC2 by Western blot analysis in HT29 and MDA‐MB231 cells. (C) Reversed IP after IP of HDAC2 and detection of SCD1 by Western blot analysis in HT29 and MDA‐MB231 cells. (D) Correlation analysis of *SCD1* gene (SCD) expression with that of other genes revealed a correlation score of 0.496 with that of *HDAC2* in all cancers (*p* = 0.001005). (E and F) Scatter plot showing the relationships between the genome‐wide correlations of the coexpression of *SCD1* (SCD) and *HDAC2* in all human cancer tissues (E) and in breast cancer tissues (F). Displayed *R* values were determined by Pearson correlation. (G) Top differentially (left) and similarly (right) correlated pathways between *SCD1* (SCD) and *HDAC2* target genes in all cancer types. All experiments were reproduced at least three times (*n* = 3), and correlation analysis was performed using ARCHS4 v8 (Feb 2020), as shown in Figure 5.

To determine whether SCD1‐associated NPM1 acetylation involves deacetylases, we examined SIRT1, SIRT3, and SIRT7 but found no differences in expression between control and SCD‐KO cells (Figure S7A–C). The acetyltransferase EP300 levels were also unchanged (Figure S7D), and inhibition of EP300 reduced NPM1 acetylation in both control and SCD‐KO cells (Figure S7E), suggesting that SCD1 regulates NPM1 acetylation via a deacetylase other than EP300. To determine whether SCD1‐associated deacetylation relies on the recruitment of histone deacetylases (HDACs), we first analyzed the mRNA expression of HDAC family members (HDAC1–8) by qRT‐PCR (Figure S7F,G). No significant differences in HDAC mRNA expression were detected, except for the downregulation of HDAC2 and HDAC4 expression in PLIN2‐ and SCD‐depleted HT29 cells (Figure S7F). However, HDAC2 and HDAC4 expression was not affected in PLIN2‐ or SCD1‐depleted MDA‐MB231 cells (Figure S7G). Western blot analysis (Figure 6A) revealed no differences in the protein levels of HDAC1, HDAC3, and HDAC4, whereas HDAC6 was not expressed in MDA‐MB231 and HT29 cells. A slight increase in HDAC2 levels was observed in SCD‐KO cells (MDA‐MB231 and HT29), suggesting a potential compensatory mechanism in response to SCD1 depletion. Furthermore, IP of SCD1 revealed that HDAC2 was present in SCD1‐containing complexes in HT29 and MDA‐MB231 cells (Figure 6B), which was further validated by reverse IP in these cells (Figure 6C). In contrast, HDAC3, HDAC4, and HDAC6 were not detected in the SCD1 immunoprecipitates from MDA‐MB231 and HT29 cells, although a faint HDAC3 signal was also observed in the IgG control (Figure S8A). Interestingly, the *SCD* and *HDAC2* genes were strongly correlated (0.496) across various cancer types (*p* = 0.00105), including breast cancer (correlation score *=* 0.506; *p* = 0.006018) (Figure 6D–F and Figure S8B). In addition, pathway analysis highlighted common target genes and pathways associated with the *SCD* and *HDAC2* genes in various human cancer types, including breast cancer (Figure 6G and Figure S8C–E). Together, these data highlight the functional role of SCD1 and HDAC2 in posttranslational protein modification in cancer.

### 2.7 The Inhibition of SCD1 Sensitizes Cancer Cells to HDAC Inhibitors

**FIGURE 7:**
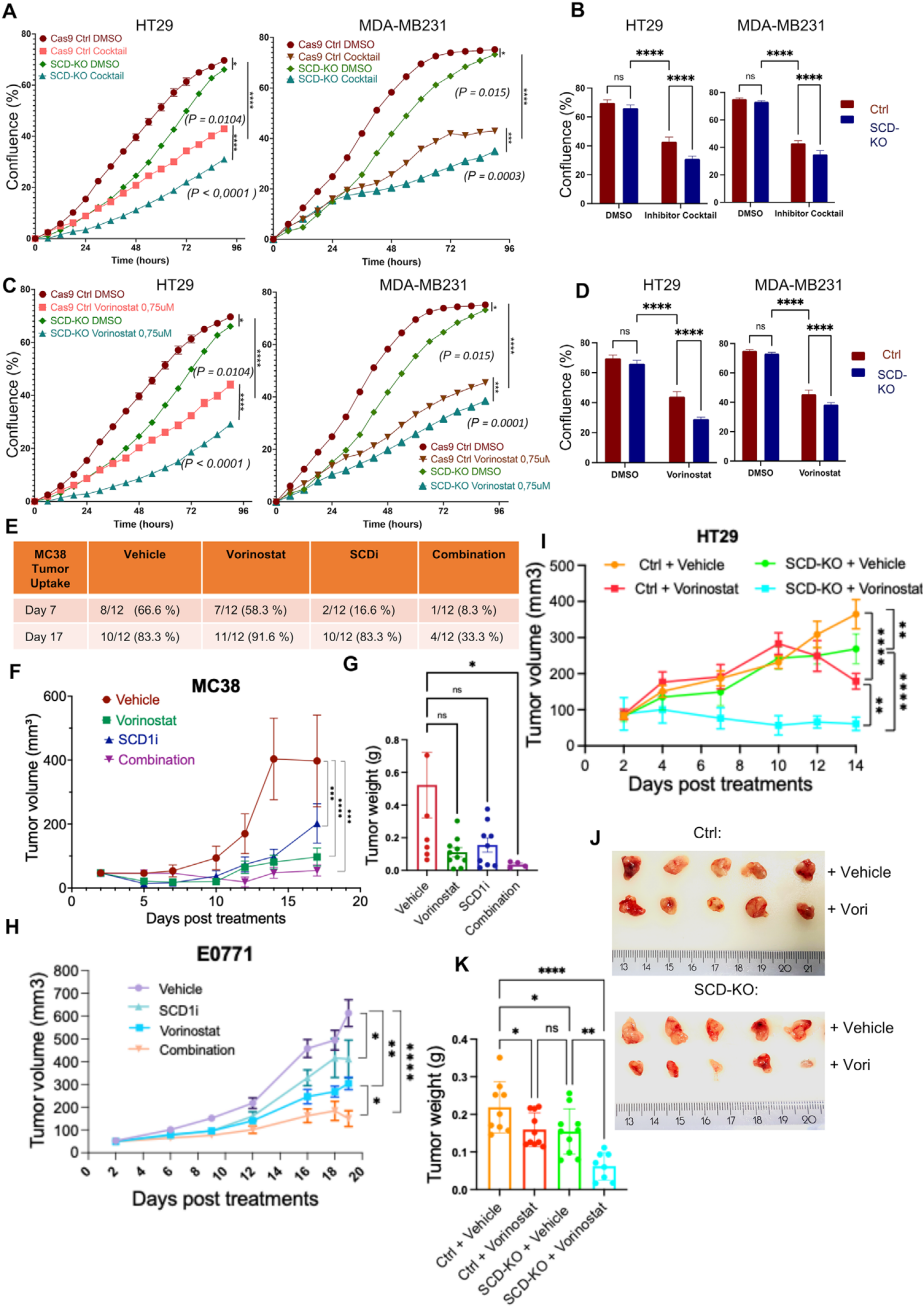
SCD‐KO cells are sensitive to acetylation inhibitors. (A) Proliferation assay with *Incucyte* at the indicated time points in control, SCD‐KO HT29 and MDA‐MB231 cells treated with DMSO (Ctrl) or a deacetylase inhibitor cocktail. (B) Cell proliferation assessment after 96 h of treatment. (C) Proliferation of cells treated with DMSO (Ctrl) or vorinostat (0.75 µM). (D) Cell proliferation assessment of cells treated as described in (C) at 96 h. (E) Tumor uptake table of MC38 mouse colorectal tumors on Day 7 and Day 17 post‐injection in C57/Bl6 mice with different treatments. (F) Growth of MC38 tumors in C57/Bl6 mice and treated with vehicle, vorinostat, or an SCD inhibitor (SCD1i) alone or with the combination of vorinostat and anti‐SCDi. (G) Tumor weights of MC38 tumors. (H) Growth of E0771 tumors in C57BL/6 mice treated with vehicle, vorinostat, or an SCD inhibitor (SCD1i) alone or with a combination of vorinostat and SCD1i. (I) Growth of HT29 control (Ctrl) or PLIN2‐KO (PLIN2‐KO1) cells treated with vehicle or vorinostat. HT29 tumors were injected into RAG1^−/−^ mice (*n* = 5). (J) Representative images of tumors from the experiment shown in (I). (K) Tumor weights of HT29 control and PLIN2‐KO cells treated with vehicle or vorinostat, corresponding to (I). ns: *p* > 0.05, ^*^ *p* < 0.05, ^**^ *p* < 0.01, ^***^ *p* < 0.001, and ^****^ *p* < 0.0001.

We hypothesized that SCD1 depletion could increase cancer cell sensitivity to HDACi. To test this hypothesis, we evaluated the effect of a pan‐HDACi cocktail on the proliferation of SCD‐KO and WT cells over 96 h using the *Incucyte* system (Figure 7A,B). All parental cells were sensitive to the HDACi cocktail. Notably, depletion of SCD1 in HT29 and MDA‐MB231 cells resulted in delayed tumor cell proliferation (over 96 h), with these cells exhibiting significantly increased sensitivity to the HDACi cocktail (*p* < 0.001 and *p* < 0.0001, respectively) (Figure 7A). Compared with the control and single agent treatment, the combination of SCD1 depletion and the HDACi cocktail was more effective at 96 h (Figure 7B). Similar results were observed in vitro with vorinostat, an FDA‐approved HDACi (Figure 7C,D). Specifically, compared with their parental counterparts, SCD1‐depleted HT29 (*p* < 0.0001) and MDA‐MB231 (*p* = 0.0001) cells exhibited greater inhibition of proliferation by vorinostat (Figure 7C). Compared with the control treatment and single agent treatment, the combination of SCD1 depletion and vorinostat was more effective at 96 h (Figure 7D).

To investigate the in vivo efficacy of this combination therapy, we utilized the MC38 syngeneic colorectal mouse model. Tumor‐bearing mice were treated with vehicle, an SCD1i, vorinostat, or a combination of both agents (Figure 7E,F). The incidence of measurable tumors at 7 and 17 days post‐injection was markedly reduced in the combination group (Figure 7E), with only 4 tumors out of 12 injections compared with 10/12 in the vehicle group at 17 days, 11/12 in the vorinostat group, and 10/12 in the SCD1i group. The tumor volume in the MC38 model was significantly reduced by single‐agent treatment and was even more profoundly reduced by the combined treatment with SCD1i and vorinostat (Figure 7F). Compared with vehicle, only the combination therapy significantly reduced tumor weight (Figure 7G). Importantly, the combination treatment did not induce significant weight loss, indicating a tolerable safety profile (Figure S9A). Given the high immunogenicity and occasional spontaneous regression of MC38 tumors, we sought to validate the combination in a second, less immunogenic syngeneic E0771 model of triple‐negative breast cancer in C57BL/6 mice (Figure 7H). Both the SCD1i and vorinostat alone significantly reduced tumor growth; however, the combination produced a markedly stronger antitumor effect, demonstrating enhanced efficacy in an additional immunocompetent context. As observed in the MC38 model, neither SCD1 inhibition nor vorinostat alone or in combination affected mouse body weight or induced signs of toxicity (Figure S9B). To explore whether this combination strategy is effective in human tumor xenografts, we implanted HT29 control and SCD‐KO cells into immunodeficient RAG1^−/−^ mice. Once the tumors reached ∼75 mm^3^, the mice were treated with vehicle or vorinostat (Figure 7I). Control HT29 xenografts showed a modest but significant response to vorinostat beginning on Day 14. Strikingly, compared with those predicted from in vitro data, SCD‐KO xenografts were highly sensitive to vorinostat in vivo and exhibited far stronger suppression, culminating in the nearly complete inhibition of tumor growth (Figure 7I). As shown in representative images (Figure 7J), tumor size and tumor weight (Figure 7K) were markedly lower in the SCD‐KO + vorinostat group than in the vehicle group or in the groups receiving single treatment. Like in the syngeneic models, vorinostat treatment did not affect mouse body weight (Figure S9C).

Finally, because both vorinostat and SCD1 inhibition can induce metabolic stress, we assessed lipid peroxidation following treatment in vitro (Figure S9D,E). As expected, SCD1 inhibition increased lipid peroxidation, as detected by C11‐BODIPY fluorescence by FACS analysis. Notably, vorinostat treatment resulted in a measurable increase in lipid peroxides, indicating that HDAC inhibition contributes to oxidative lipid damage. To determine whether specific inhibition of HDAC2 could reproduce the effects observed with vorinostat and whether this response would be further enhanced in the absence of SCD1 (Figure S9F,G), we treated multiple cancer cell lines (MDA‐MB‐231, HT29, and HS578T) with santacruzamate A (HDAC2i). HDAC2 inhibition significantly reduced cancer cell proliferation and occurred more effectively in SCD1‐depleted cells than in control cells. These results validate HDAC2 and SCD1 as cooperative therapeutic targets and support the concept that dual interference with HDAC2 activity and lipid desaturation produces a stronger antiproliferative effect.

Collectively, these data indicate that SCD1 inhibition markedly enhances tumor sensitivity to HDACi across multiple in vivo models, supporting a compelling therapeutic strategy for suppressing tumor growth.

## 3 Discussion

In recent years, the effects of lipid metabolism on cancer progression and therapeutic response have attracted increasing attention [17, 43, 44, 45, 46, 47]. Here, we provide evidence linking tumor hypoxia to altered lipid metabolism, with downstream effects on protein regulation and cancer cell behavior. We show that hypoxia induces the expression of SCD1 and PLIN2, promoting LD accumulation. These proteins exhibit a reciprocal regulatory relationship: PLIN2 promotes cancer cell survival under hypoxia, whereas SCD1 primarily regulates proliferation and migration under normoxic and reoxygenation conditions. This interplay suggests that cancer cells dynamically coordinate lipid desaturation and storage to limit lipid peroxidation and sustain survival under stress.

SCD1 plays a central role in maintaining the balance between SFAs/MUFAs, thus protecting cells from oxidative stress and ferroptosis [20, 27]. Consistent with our previous findings [25], SCD1 limits reactive oxygen species (ROS) and ferroptotic sensitivity by enriching membranes with MUFAs and reducing polyunsaturated fatty acids (PUFAs), particularly during the transition from hypoxia to reoxygenation. In the present study, we extend these observations by demonstrating that SCD1 also regulates nonhistone protein acetylation, suggesting a broader role in coordinating metabolic and posttranslational responses to stress.

Interestingly, depletion of SCD1 or PLIN2 led to distinct effects on protein acetylation. Both conditions increased intracellular acetyl‐CoA levels more prominently in SCD1‐deficient cells. This was accompanied by reduced expression of acetyl‐CoA carboxylase‐1 (ACC‐1), indicating decreased de novo lipogenesis and accumulation of acetyl‐CoA. However, global acetylome profiling did not reveal a uniform increase in protein acetylation. Instead, subsets of proteins were either hyperacetylated or deacetylated depending on the presence of SCD1 or PLIN2. Overall, PLIN2 was associated with increased acetylation of specific targets, whereas SCD1 promoted their deacetylation, suggesting that a reversible and selective regulatory mechanism may facilitate cellular adaptation to metabolic stress.

Among the proteins regulated by SCD1‐dependent deacetylation, we identified NPM1, a multifunctional chaperone implicated in tumor suppression that is frequently altered in cancer [37, 48, 49] and that is commonly mutated, rearranged and deleted in several cancer types [37, 38, 40, 48, 50, 51, 52]. We showed that SCD1 promotes the deacetylation of NPM1, affecting its stability and subcellular localization between the nucleus and cytoplasm, which may influence its roles in cell cycle regulation and tumor progression [50, 53, 54]. This pathway is further supported by strong correlations between *SCD1* and *NPM1* target gene expression in human cancers, suggesting the existence of a functional signaling axis linked to malignancy.

Mechanistically, we provide evidence that SCD1 may recruit HDAC2 to form a molecular complex with NPM1, thus facilitating the deacetylation of NPM1. Consistently, gene expression analyses revealed strong correlations between SCD1, NPM1, and HDAC2 across human tumors, supporting a coordinated regulatory network. These findings suggest that SCD1 not only controls lipid metabolism but also contributes to transcriptional and posttranslational regulation through modulation of protein acetylation. This finding expands the functional scope of SCD1 and highlights its potential role at the interface between metabolic and epigenetic regulation.

Given the limited efficacy of HDACi in clinical settings [55, 56, 57, 58], we explored whether targeting SCD1 could enhance their therapeutic impact. Our in vitro data demonstrate that compared with single treatments, combined inhibition of SCD1 and HDAC significantly reduces cancer cell proliferation. This effect was confirmed in vivo using an MC38 colorectal cancer model, in which SCD1 inhibition potentiated the antitumor activity of the HDACi vorinostat [59]. Although MC38 tumors are highly immunogenic and may undergo spontaneous regression, we validated these findings in a less immunogenic syngeneic model (E0771) and in human HT29 xenografts, including those with SCD‐KO derivatives. The enhanced response in immunodeficient mice indicates that the observed synergy is tumor cell–intrinsic rather than immune‐mediated. Notably, the magnitude of tumor suppression in vivo exceeded that observed in vitro, suggesting that SCD1 inhibition may additionally impact tumor microenvironmental factors such as metabolic stress or nutrient availability. Mechanistically, both SCD1 inhibition and vorinostat treatment increased lipid peroxidation, which is consistent with enhanced oxidative and metabolic stress. HDAC inhibition alone elevated lipid peroxide levels, in line with its known capacity to increase ROS levels [60]. Furthermore, the selective inhibition of HDAC2 using santacruzamate A recapitulated the effects of vorinostat and resulted in stronger antiproliferative effects in SCD1‐deficient cells. These findings identify SCD1 and HDAC2 as cooperative vulnerabilities, and their combined targeting amplifies metabolic and epigenetic stress, resulting in enhanced tumor suppression.

## 4 Conclusions

Our study identifies SCD1 and PLIN2 as key mediators of cancer cell adaptation to hypoxia through the coordinated regulation of lipid metabolism and protein acetylation. SCD1 promotes deacetylation and stabilizes the tumor suppressor NPM1 via a complex involving SCD1, HDAC2, and NPM1. Importantly, we demonstrate that targeting SCD1 enhances the efficacy of HDAC inhibition, providing a rationale for combination therapeutic strategies. Future studies in human tumor samples will be essential to validate these findings and assess their clinical relevance.

## 5 Materials and Methods

### 5.1 Cell Lines and Chemicals

Human triple‐negative breast cancer cells (MDA‐MB231 and HS578T) and human colorectal cancer cells (HT29 and HCT116) were purchased from American Type Culture Collection (ATCC) (Manassas, USA). The murine colon adenocarcinoma (MC38) cell line was obtained from Sigma‐Aldrich (Merck, USA), and the murine triple‐negative breast cancer (E0771) cell line was obtained from CH3 BioSystems (Amherst, New York, USA) and the Lewis lung carcinoma (LLC) cells from (ATCC). CRISPR‐Cas9 stable cell lines for human breast cancer (MDA‐MB231 and HS578T) and human colorectal cancer (HT29 and HCT116) were purchased from GeneCopoeia (Maryland, USA). The cell lines were tested with a MycoALert Kit 139 (Lonza) to ensure that they were *Mycoplasma* free before the in vitro and in vivo experiments were performed. Cell line authentication, including verification for interspecies contamination, is regularly performed by STR analysis at the Leibniz Institute DSMZ GmbH (Braunschweig, Germany). MDA‐MB231, HS578T, HCT116, MC38, and LLC cells were grown in Dulbecco's modified Eagle's medium (DMEM), and HT29 and E0771 cells were cultured in RPMI basal medium. The culture medium was supplemented with 10% fetal bovine serum (FBS), L‐glutamine (2 mM), penicillin (100 U/mL), and streptomycin (100 µg/mL). All the cell culture reagents media used were from Thermo Fisher (Waltham, USA). Cycloheximid (#C4859) and mitomycin (#M4289) were obtained from Sigma‐Aldrich (St. Louis, USA). The potent and orally bioavailable SCD1i A939572 was purchased from Abcam (#Ab142089; Abcam, Cambridge, UK) for in vitro study and from TargetMol for in vivo study (#T4534; TargetMol, Boston, USA). The LD detection agent Bodipy 493/503 (# HY‐W090090) and the deacetylase inhibitor cocktail (# HY‐K0030) were purchased from MediChemExpress (Monmouth Junction, USA). Vorinostat (#V‐8477) was purchased from LC Laboratories (Woburn, USA). Santacruzamate A (CAY10683; MedChemExpress) and SGC‐CBP30 (Sigma‐Aldrich, Merck) were used as HDAC2 and EP300 inhibitors, respectively.

### 5.2 Mouse Models and Drug Administration

All animal procedures were performed in accordance with the Federation of European Laboratory Animal Science Associations (FELASA) guidelines within the accredited institutional animal facility at Liège University, Belgium, under approved animal protocols #1990, #1991, and #2458. In vivo tumor growth studies were conducted on MDA‐MB231 and HT29 xenografts from immunodeficient nude RAG2^−/−^ mice (6–8 weeks old) (Envigo). The mice were injected subcutaneously (sc) with 1.10^6^ cells of either SCD‐KO MDA‐MB231 or HT29 cells, along with their respective WT control cells (*n* = 6). Mouse health was monitored daily, and caliper measurements of tumor size commenced once the tumors became palpable. Syngeneic mouse tumors derived from MC38 or E0771 cells were generated in 6‐ to 8‐week‐old female C57BL/6 mice (Janvier). The mice were injected sc with 1 × 10^5^ cells for MC38 or 0.5 × 10^6^ cells for E0771, and when the tumor volume reached 50–75 mm^3^, the mice were randomized into four treatment groups. In the SCD1i group, the mice were treated via gavage with 40 mg/kg/day SCD1i dissolved in vehicle‐1 (2.5% DMSO, 0.5% methylcellulose, 0.25% Tween 80 in H_2_O) 5 days/week. In the vorinostat group, the mice received intraperitoneal (ip) injections of vorinostat at 100 mg/kg/day dissolved in vehicle‐2 (2.5% DMSO, 30% PEG300, 0.25% Tween 80 in H_2_O). In the combination group, the mice were treated with SCD1i by gavage and vorinostat by ip injection. Control mice received vehicle‐1 for SCD1i and vehicle‐2 for vorinostat. Tumor sizes were measured twice weekly as previously described [25], and body weights were recorded twice weekly. For the pharmacological studies, 6 mice per group (*n* = 6) were used for MC38 and E0771 tumors, and 5 mice per group (*n* = 5) were used for the HT29 control and SCD‐KO tumors grown in RAG1^−/−^ mice (Animal facility, CHU de Liège). Data were analyzed using Excel for the calculation of tumor volumes, and GraphPad Prism was used for statistical analyses. Please refer to the statistical analysis section for details.

### 5.3 PLIN2 and SCD1 Depletion

PLIN2‐ and SCD1‐KO (PLIN2‐KO and SCD‐KO) cells were generated by CRISPR Cas9 technology. MDA‐MB231 (#SL515) and HT29 (#SL523) cell lines stably expressing CRISPR‐Cas9 nuclease were obtained after transgene transduction and selection by hygromycin at 500 mg/mL for MDA‐MB231 cells and 1 mg/mL for HT29 cells (GeneCopoeia, Maryland, USA). CRISPR‐cas9‐expressing MDA‐MB‐231 and HT29 cells were transduced with a pool of either 3 PLIN2 or SCD1 gRNAs as recommended by GeneCopoeia. Parental HS578T and HCT116 SCD‐KO cells, in which either SCD1 or PLIN2 was targeted, were generated using the same procedures described for HT29 and MDA‐MB‐231 cells. Clones were isolated using a colony formation assay, and the resulting KO cells were validated by Western blot analysis for SCD1 or PLIN2 expression. To deplete SCD1 by shRNA, MDA‐MB231 cells depleted of SCD1 by shRNA (SCD1‐KD) and their control (Ctrl) containing nontargeted shRNA were generated as described previously [25].

### 5.4 Western Blot Analysis

Cell lysates were prepared as previously described [25]. Total protein extracts (50 µg) were separated under reducing conditions on 5%–15% polyacrylamide gels (depending on the molecular weight) and transferred onto polyvinylidene difluoride membranes (NEN, Boston, MA, USA). The membranes were saturated for 2 h with casein (1%, w/v) in PBS‐Tween‐20 (0.1%, v/v). Antigenic bands were detected by exposing the membranes to human primary antibodies targeting the following proteins: PLIN2 (#ab78920; Abcam), SCD1 (#ab19862; #ab236868; Abcam), NPM1 (ab15440; Abcam), acetylated‐lysine (#9814; Cell Signaling), HDAC Antibody Sampler Kit (9928; Cell Signaling), EP300 (#MA1‐16608), SIRT1 (#9475), SIRT3 (#PA5‐28402), SIRT7 (#D3K51), P21 (#ab109199), lactate dehydrogenase (#ab101562), CD44 (#ab157107; Abcam), filamin A (#ab11074), PSMA7 (#5688‐M01; Abnova), FASN (#3180S), phospho‐ACC1 (#3661; Cell Signaling), ACC1 (#3662; Cell Signaling), and CPT1 (#ab53532). After they were washed, the membranes were incubated with a secondary horseradish peroxidase (HRP)‐conjugated goat anti‐rabbit antibody (1:2000, Dako Cytomation) or a sheep anti‐mouse antibody (1:1000, Dako Cytomation). Immunocomplexes were visualized by chemiluminescence on a luminescent image analyzer (Amersham ImageQuant800). For loading control, the membranes were stripped and reincubated with Hsc70 (#B‐6, sc‐7298) antibody (Santa Cruz, CA, USA) or with GAPDH (#MAB37A) and actin (#A2066) antibody (Sigma‐Aldrich, St. Louis, USA).

### 5.5 Coimmunoprecipitation Assay

Cell lysates were lysed in lysis buffer (Cell Signaling Technology) containing a protease inhibitor cocktail (cOmplete, Roche), a phosphatase inhibitor cocktail (PhosSTOP, Roche) and a deacetylase inhibitor cocktail (#HY‐K0030, MedChemExpress). The protein was immunoprecipitated from lysates (1 mg) using the appropriate antibody (1 µg) and 50 µL of 50% slurry protein G coupled to Dynabeads (Invitrogen). The precipitate was subjected to Western blotting using a rabbit monoclonal anti‐NPM1 antibody (#ab15440; Abcam), anti‐SCD1 antibody (#ab19862; Abcam), and anti‐HDAC antibody (#9928; Cell Signaling). The antibodies were revealed with an HRP‐conjugated secondary antibody.

### 5.6 Proximity Ligation Assay

PLAs in cancer cells in vitro were performed following the protocol previously described by Pacchiana et al. [61] on HT29 and MDA‐MB231 cells. Coverslips were washed with PBS, fixed with 4% PFA and permeabilized with 0.25% Triton X‐100 for 10 min at RT, after which they were washed with PBS and incubated with Duolink blocking solution for 30 min at 37°C. The cells were incubated with anti‐NPM1 antibody (ab15440; Abcam) and anti‐SCD1 antibody (ab19862; abcam) at 1/100 dilution overnight at 4°C. After they were washed, the cells were incubated with secondary antibodies (Duolink In Situ PLA Probe Anti‐Rabbit PLUS DUO92002, Anti‐Mouse MINUS, DUO92004, Sigma‐Aldrich) coupled with oligonucleotides at 1/40 for 2 h at 37°C. After they were washed, the cells were incubated in Duolink ligation solution (1/40 enzyme, 1/5 buffer) (Duolink In Situ Detection Reagents Red, DU92008, Sigma‐Aldrich) for 15 min at 37°C, after which they were washed and incubated in Duolink amplification solution (1/80 enzyme, 1/5 buffer) for 90 min at 37°C. Slides were mounted with DAPI Fluoromount G (0100–20; Southern Biothec) and analyzed by fluorescence microscopy (Olympus, Zeiss HR LSM 880).

### 5.7 Immunohistochemistry Analysis of Tumor Sections

Paraffin‐embedded tumor sections were processed for immunohistochemistry (IHC). Antigen retrieval was performed for 11 min at 126°C (Dako Target Retrieval Solution, S2031), followed by 20 min blocking of endogenous peroxidase (3% H_2_O_2_) and 20 min blocking with animal‐free solution (Cell Signaling, 15019L). For CAIX and SCD1, sections were incubated overnight at 4°C with anti‐CAIX (Abcam, Ab15086; 1:1000) or anti‐SCD1 (Abcam, Ab19862; 1:100), followed by HRP‐based detection with TSA amplification (FITC for CAIX, Cy5 for SCD1). Ki67 and cleaved caspase‐3 staining followed a previously described protocol [62]. Briefly, Ki67 was detected using anti‐Ki67 (Abcam, Ab833; 1:500) with EnVision/HRP (Dako, K4003). Cleaved caspase‐3 (Cell Signaling, 9661L; 1:300) was incubated overnight at 4°C and detected with EnVision/HRP and DAB. Slides were scanned using a Hamamatsu NanoZoomer system, and Ki67 and caspase‐3 quantification was performed in QuPath v3.2 following official guidelines.

### 5.8 Detection of Lipid Peroxidation by Flow Cytometry

HT29 and MDA‐MB‐231 cells were seeded in triplicate in 24‐well plates for 24 h. The cells were then treated with DMSO, the SCD1i A939572 (20 µM), vorinostat (0.75 µM), or both inhibitors for 24 h. Subsequently, the cells were incubated with 1 µM BODIPY 581/591 C11 (Invitrogen) at 37°C for 30 min. After trypsinization, the cells were washed with FACS buffer by centrifugation, and the fluorescence intensity of the cells with BODIPY 581/591 C11 staining was measured by flow cytometry. The fold change in the median fluorescence intensity (MFI) over the control group (Ctrl) was then calculated for each sample. All flow cytometry analyses were performed using a CytoFLEX flow cytometer (Beckman Coulter Life Sciences) after which the data were analyzed, and images were captured by FlowJo software (BD Biosciences).

### 5.9 Gene Coexpression Correlation Analysis

Gene correlation and discovery with the *HIF1a*, *SCD*, *NPM1*, and *HDAC2* genes were analyzed by the *Correlation AnalyzeR* tool described previously [63]. The interface used and the database are available as a web application at https://gccri.bishop‐lab.uthscsa.edu/correlation‐analyzer/and as a standalone R package at https://github.com/Bishop‐Laboratory/correlationAnalyzeR.

### 5.10 Statistical Analysis

For in vitro experimentation, differences between experimental groups were assessed using a Mann‒Whitney test with Prism 7.0/8.0 software (GraphPad, San Diego, CA). For computerized image analysis, statistical analysis was performed using the statistical toolbox of MATLAB (9.4) 9 (R2018b) software (MathWorks, Inc.). The results are expressed as the mean ± standard error of the mean (SEM). A nonparametric Mann‒Whitney significance test was used to compare the median values measured for the groups. The results were considered significant at *p* < 0.05. In vivo data were analyzed using GraphPad Prism 8. Pairwise comparisons between the vehicle‐treated and drug‐treated cohorts were performed using a two‐tailed Student's *t*‐test. Longitudinal tumor growth data were evaluated using two‐way repeated‐measures ANOVA to account for within‐subject measurements over time. Endpoint analyses involving all four treatment conditions were conducted using one‐way ANOVA.

## Author Contributions

N.E.S. and C.W. conceptualized and designed the study. C.W., L.M.R., E.B., M.F., R.J., Q.H.G., Y.B., O.P., M.F., G.F., F.D., and N.E.S. developed the methodology. C.P., L.M.R., E.B., Q.H.G., G.C., Y.B., L.M.R., and G.F. acquired the data. C.W., L.M.R., E.B., M.F., R.J., C.P., S.CB, G.F., M.F., O.P., and N.E.S. analyzed and interpreted the data (e.g., statistical analysis, biostatistics). N.E.S., O.P., A.N., and C.W. wrote, reviewed, and/or revised the manuscript. N.E.S. supervised the study, acquired funding, and administered the project. All authors have read and approved the final manuscript.

## Ethics Statement

All animal procedures complied with FELASA guidelines and European Directive 2010/63/EU and were conducted in the accredited animal facility at the University of Liège, Belgium, under approved protocols #1990, #1991, and #2458 (approved February 28, March 30, and June 30, 2022, respectively).

## Conflicts of Interest

The authors declare no conflicts of interest.

## Supporting information




**Supplementary Materials and Methods** include cell proliferation, migration, immunofluorescence, gPCA, metabolic flux analysis (OCR/ECAR), qRT–PCR, acetyl‐CoA quantification (UHPLC–ESI–MS/MS), and LC–MS acetylomics
**Supporting Information**,
Additional supporting information is available online. This includes supplementary materials and methods, as well as nine supplementary figures (Figures S1–S9).

## Data Availability

The data that support the findings of this study are available from the corresponding author upon reasonable request. The proteomic data were deposited in PRIDE with accession number: PDX077399.
