# Supplementary material for: Stearoyl‐CoA Desaturase‐1 Drives Tumor Growth by Interacting With Histone Deacetylase‐2 and Deacetylating Nucleophosmin‐1
Source: MedComm (2020). 2026 Jun 11;7(6):e70809. doi: 10.1002/mco2.70809 (PMC13260692; doi:10.1002/mco2.70809)
Supplement: Supplementary file 1 — Supplementary Materials and Methods include cell proliferation, migration, immunofluorescence, gPCA, metabolic flux analysis (OCR/ECAR), qRT–PCR, acetyl‐CoA quantification (UHPLC–ESI–MS/MS), and LC–MS acetylomics Supporting Information, Additional supporting information is available online. This includes supplementary materials and methods, as well as nine supplementary figures (Figures S1–S9). [file MCO2-7-e70809-s001.pdf]

Supplementary data file

Stearoyl-CoA desaturase-1 drives tumor growth by interacting with histone deacetylase-2 and deacetylating nucleophosmin-1

Authors: Coline Wery[1], Laetitia Montero-Ruiz[1,2], Eric Bonneil[4], Mohammad Farran[1], Robin Jehay[1], Quentin Herrara Garfia[1], Gregory Fettweis[3], Silvia Blacher[2], Charles Pottier[1], Gael Cobraiville[5], Yasmine Boumahd[6], Olivier Peulen[6], Agnès Noël[2], Franck Dequiedt[3], Marianne Fillet[5] and Nor Eddine Sounni[1] *

Affiliations:

[1] Cancer Metabolism and Tumor Microenvironment Lab, GIGA-Cancer, GIGA Institute, University of Liège, Liège, Belgium.

[2] Laboratory of Tumor and Development Biology, GIGA Institute, University of Liège, Liège, Belgium

[3] Laboratory of Gene Expression and Cancer, GIGA Institute, University of Liège, 4000 Liège, Belgium

[4] Institute for Research in Immunology and Cancer (IRIC), University of Montréal, Montréal, Canada

[5] Laboratory for the Analysis of Medicines, CIRM Institute, University of Liège, Liège, Belgium

[6] Metastasis Research Laboratory, GIGA-Cancer, GIGA Institute, University of Liège, Liège, Belgium

Running title: *SCD1 as a dual regulator of cancer survival and sensitivity to therapy*

Keywords: Cancer drug resistance; HDACs; Lipid droplets; Non-histone protein deacetylation NPM1; SCD1

**Corresponding author. Email: nesounni@uliege.be*

Supplementary materials and methods

Supplementary Figures S1-9

Supplementary materials and methods.

5.11 Cell proliferation

Cell proliferation was assessed with the CYQUANT assay according to the manufacture's protocol. For cell proliferation assessment with the *Incucyte*®, HT29 (1×10^4) and MDA-MB231 (1×10^4) cells were plated in 96 well-plates with DMEM 10% FBS. Data of proliferating profiles were collected and processed from *Incucyte*® (Sartorius, Göttingen, Germany). For hypoxic conditions and cell proliferation measurements, images were aquired at the start of the experiments and after 24 hours of hypoxic incubation. For treatments, cells were incubated either with DMSO (2%), with vorinostat (#V-8477, LC Laboratories, Woburn, USA), santacruzamate A (# CAY10683, MedChemExpress, New Jersey, USA) or Deacetylase Inhibitor Cocktail (# HY-K0030, MediChemExpress, Monmouth Junction, USA).

5.12 Cell migration

For cell migration assessment with the *Incucyte*®, HT29 ($4,5\times10^3$) and MDA-MB231 ($4,5\times10^3$) cells were plated in 96 well-plates with DMEM 10 % FBS supplemented with mitomycin. Once cells are attached on the plate, a scratch is performed with the WoundMaker according to the manufacturer's protocol. Data of proliferating profiles were collected and processed from *Incucyte*® (Sartorius, Göttingen, Germany). For hypoxic conditions and cell migration measurements, images were aquired at the start of the experiments and after 24 hours of hypoxic incubation.

5.13 Immunofluorescence analysis

Immunofluorescence detection was performed on coverslips of cancer cells in vitro, including Control and knockout (KO) MDA-MB-231 and HT-29 cells. For PLIN2 analysis, cells were incubated under normoxic or hypoxic conditions (1% O_2) for 24 h. For NPM1 and lipid droplet detection, cells were maintained under standard culture conditions. Coverslips were washed with PBS, fixed in 4% paraformaldehyde, and (for protein detection) permeabilized with 0.5%

Triton X-100 for 10 min at room temperature (RT). After washing, samples were blocked with 5% BSA for 30 min at RT. For protein detection, cells were incubated overnight at 4°C with primary antibodies diluted (1:100) in 1% BSA/PBS: anti-PLIN2 (ab78920, Abcam) or anti-NPM1 (MABE938, Sigma-Aldrich). After washing, cells were incubated with donkey anti-rabbit Alexa Fluor 555 secondary antibody (A31572, Invitrogen; 1:200) for 1h at RT. For lipid droplet staining, fixed cells were incubated with BODIPY 493/503 for 20 min at RT, followed by PBS washes. All samples were mounted with DAPI Fluoromount-G (0100–20, SouthernBiotech) and imaged using fluorescence microscopy (Olympus; Zeiss LSM 880).

5.14 Gaussia protein-fragment complementation assay (gPCA)

To investigate the interaction between SCD1 and NPM1, open reading frames (ORFs) were obtained from the hORFeome 7.1 and 8.1 collections. ORFs were amplified by PCR and cloned into the pDONR223 entry vector via BP recombination using Gateway technology (Invitrogen). All clones were verified by sequencing. Entry clones were then recombined into destination vectors encoding the GLucN1 and GLucN2 fragments of Gaussia princeps luciferase using LR Clonase reaction. gPCA assays were carried out as previously described in [42]. Briefly, HEK293T cells were seeded in 24-well plates and transfected with the appropriate constructs. After 48 hours, cells were washed with PBS, and lysates were prepared following the manufacturer's instructions (Renilla Luciferase Kit, Promega). Luminescence was measured in triplicate using a TriStar 2 S LB 942 luminometer (Berthold). Experiments were performed with at least three independent biological replicates per construct. Normalized luminescence ratios (NLR) were calculated as: $\mathrm{NLR} = \frac{\text{signal (Glu1A + Glu2B)}}{\text{signal (Glu1A + Glu2) + signal (Glu1 + Glu2B)}}$ where **A** and **B** represent the proteins of interest, **Glu1** and **Glu2** are the split luciferase fragments alone, and **Glu1A/Glu2B** are the fragments fused to proteins A and B, respectively. Interactions were

evaluated using a one-tailed one-sample t-test against a value of 3.5, as described by Cassonnet et al. [41] . Positive interactions were defined at $p < 0.05$.

5.15 Measurement of oxygen consumption and extracellular acidification rates

Oxygen consumption rate (OCR) was measured using the Extracellular consumption assay from Abcam (#ab197243) as instructed by the fabricant. Fatty acid Oxidation rate (FAO) was measured using the Fatty Acid Oxidation Assay from Abcam (#ab217602) as instructed by the manufacturer. Kinetic profiles of oxygen consumption rates (OCR) and extracellular acidification rate (ECAR) were obtained using a Seahorse XFp Extracellular Flux Analyzer (Agilent) according to the manufacturer's protocol. Cells were seeded in normal growth medium 40 h before measurement at a density of $17{,}5\times10^3$ cells per well for MDA-MB231 cells and 2×10^4 per well for HT29 cells. Cells were first incubated in normal conditions overnight then 24h in a hypoxic incubator with 1% of oxygen. One hour before the assay, cells were incubated in unbuffered serum-free DMEM (Basal DMEM, Agilent) pH 7.4, supplemented with 10 mM glucose, 1 mM sodium pyruvate and 2 mM glutamine at 37°C in a non-CO2 incubator. Measurements of OCR and ECAR were performed before and after sequential injection of inhibitors. The glucose pathway is inhibited by UK5099 (2 μM) targeting the mitochondrial pyruvate carrier. The glutamine pathway is inhibited by BPTES (3 μM) targeting the glutaminase-1 and the fatty acid pathway is inhibited by etomoxir (4 μM) targeting the carnitine palmitoyl-transferase-1A. The cell dependence to fatty acids is measured by the sequential inhibition induced by UK5099 and BPTES, followed by Etomoxir. The cell capacity to use fatty acids is measured by the sequential inhibition induced by Etomoxir, followed by UK5099 and BPTES. The flexibility is calculated as the subtraction of the dependence from the capacity. The data were normalized after cell counting with Hoechst staining.

5.16 RNA extraction and qRT-PCR analysis

RNA was extracted using the High Pure RNA Isolation Kit (Roche Diagnostics Applied Science, Mannheim, Germany) or RNeasy Mini kit (Qiagen GmbH, Hilde, Germany) for cells, according to manufacturer's recommendations. RNA quality check including concentration and purity was performed with Nanodrop One (Thermo Scientific). For the QRT-PCR on cells RNA, 1 µg of RNA were used for reverse transcription with FastGene Scriptase II cDNA 5x ReadyMix, LS64, Nippon Genetics (*Düren, Germany, Europe*) and SimpliAmp™ Thermal Cycler (Thermo Fisher Scientific). Then, 10 ng of cDNA were mixed with primers Forward + Reverse (20 µmol/L) and Sybergreen (FastStart Universal SYBR Green Master (ROX) 04913914001/ NipponGenetics). The mix was analyzed with the QuantStudio 3 (appliedBiosystems - Thermo Fisher Scientific). Relative HDAC mRNAs expression was calculated from Cq values reported to GAPDH mRNA and using the ΔCq method. Primers used are listed in the Table-1.

**Target**	**Sequence**
**HDAC1**	Fw: 5' ACC GGG CAA CGT TAC GAA T 3'<br>Rv: 5' CTA TCA AAG GAC ACG CCA AGT G 3'
**HDAC2**	Fw: 5' TCA TTG GAA AAT TGA CAG CAT AGT 3'<br>Rv: 5' CAT GGT GAT GGT GTT GAA GAA G 3'
**HDAC3**	Fw: 5' TTG AGT TCT GCT CGC GTT ACA 3'<br>Rv: 5' CCC AGT TAA TGG CAA TAT CAC AGA T 3'
**HDAC4**	Fw: 5' AAT CTG AAC CAC TGC ATT TCC A 3'<br>Rv: 5' GGT GGT TAT AGG AGG TCG ACA CT 3'
**HDAC5**	Fw: 5' TTG GAG ACG TGG AGT ACC TTA CAG 3'<br>Rv: 5' GAC TAG GAC CAC ATC AGG TGA GAA C 3'
**HDAC6**	Fw: 5' TGG CTA TGG CAT GTT CAA CCA 3'<br>Rv: 5' GTC GAA GGT GAA CTG TGT TCC T 3'
**HDAC7**	Fw: 5'CTG CAT TGG AGG AAT GAA GCT 3'<br>Rv: 5'CTG GCA CAG CGG ATG TTT G 3'
**HDAC8**	Fw: 5' CAC CAT GGA GGA GCC GGA GGA A 3'<br>Rv: 5' GAC CAC ATG CTT CAG ATT CCC TTT G 3'
**SREBP1**	Fw: 5' ACT TCT GGA GGC ATC GCA AGC A 3'<br>Rv: 5' AGG TTC CAG AGG AGG CTA CAA G 3'
**SREBP2**	Fw: 5' CTC CAT TGA CTC TGA GCC AGG A 3'<br>Rv: 5' GAA TCC GTG AGC GGT CTA CCA T 3'
**SCD1**	Fw: 5' GAA GGG GAG TAC GCT AGA CTT GT 3'<br>Rv: 5' ACA TCA TCA GCA AGC CAG GT 3'
**PGC1a**	Fw: 5' CCA AAG GAT GCG CTC TCG TTCA 3'<br>Rv: 5' CGG TGT CTG TAG TGG CTT GACT 3'
**GAPDH**	FW: 5' TGT GGG CAT CAA TGG ATT TGG 3'<br>RV: 5' ACA CCA TGT ATT CCG GGT CAAT 3'

Table 1: Sequences of primers used for qRT-PCR analysis

5.17 Quantification of Acetyl-Coenzyme A by UHPLC–ESI-MS/MS

Chromatographic conditions: UHPLC was performed on a 1290 Infinity LC system coupled to a 6495 triple quadrupole mass spectrometer equipped with the iFunnel technology (Agilent Technologies, Waldbronn, Germany). Chromatographic separation was performed on a reverse-phase Kinetex Phenyl-Hexyl column (1.7 µm, 100 mm × 2.1 mm ID) protected with a Security Guard Ultra Phenyl-Hexyl precolumn (both from Phenomenex, Torrance, CA, USA). Optimal chromatographic conditions were as follows: The column compartment was thermostated at 50 °C. The separation was carried out in gradient mode with aqueous mobile phase A (50 mM NH4HCO2 and 2.5 mM TEA at pH 5.6) and organic mobile phase B (ACN/IPA, 8/2 v/v) at 0.3 ml/min. The gradient started at 3% B for 0.25 min and ramped as follows: 0.25 - 4.25 min, 90% B; 4.25 - 5.25 min; 90% B; 5.25 - 5.35 min, 3% B. The autosampler was thermostated at 10 °C and kept in the dark. One microliter of the samples was injected in triplicate for each sample. Mass spectrometric conditions: The electrospray source was operated in positive ionization mode (ESI+). The capillary and the nozzle voltage were set at 4000 V and 1100 V respectively. Nitrogen was used as dry gas and sheath gas heated at 210 °C with a flow rate of 15 l/min and 400 °C at 12 l/min, respectively. The nebulizer pressure was settled at 30 psi. The high pressure and low-pressure funnels were operated at 170 and 150 V, respectively. Fragmentation and collision energies were optimized for Acetyl-CoA. Unit mass resolution was set in both mass-resolving quadrupoles Q1 and Q3. Cell accelerator voltage was kept at 4 V. Analysis was conducted in multiple reaction monitoring (MRM) mode. Two transitions (810.1/303.1 and 810.1/136.0) were followed for Acetyl-CoA as quantifier and qualifier transitions, respectively. Collision energies were set at 30 and 50 V for transition quantifier and qualifier respectively. Calibration standards: Calibration standards were prepared with NH4HCO2 2.5 mM pH 5.6 to obtain concentrations ranging from 0.01 µM to 5 µM._Sample preparation: Acetyl-CoA was

extracted on cell pellet with 300 µl of NH4HCO2 2.5 mM pH 5.6/ACN/MeOH (1/1/1, v/v/v). Sonication was performed using a Q700 Sonicator (Qsonica, Newtown, CT, USA). Each pulse of 5 s was followed by a 5 s off pulse for a total 3 times of sonication (20% amplitude). Then samples were incubated at -20°C for 2h and centrifugation at 4 °C and 15000 x g for 20 min. The supernatants were vacuum dried for 2 h at 50 °C using a CentriVap Concentrator (LabConco, Kansas-City, MO, USA) and reconstituted in 100 µl of NH4HCO2 2.5 mM pH 5.6/ACN (8/2, v/v). The reconstituted samples were then analyzed by UHPLC–MS/MS.

Data analysis: Results were obtained with MassHunter Data Acquisition and analyzed using the Quantitative Analysis Software (Agilent Technologies, Waldbronn, Germany).

5.18 LC-MS based acetylomics

Acetylome analysis was performed by the center for advanced proteomics analyses Proteomic-IRIC Center of Montreal. Crude cell pellets of MDA-MB-231 shNT and shSCD1, CRISPR Control and CRISPR-cas9 KO PLIN2 were sent. The pellets were snap frozen in nitrogen. Cell pellets were resuspended in 5 volumes of 8 M urea 50 mM Tris-HCL pH 8.0, sonicated and centrifugated. Supernatants were quantified with Bradford assays. 5-10 mg of proteins were reduced with TCEP (1 mM), alkylated with chloroacetamide (20 mM) for 30 min at room temperature. 7 volumes of ammonium bicarbonate 50 mM were added to bring urea concentration to 1 M. Trypsin at 1:20 (enz/prot) was added and the samples were incubated overnight at 37°C. Peptide samples were acidified with TFA (1%). Samples were desalted on stage tips and dried down with a speed-vac. Samples were then resolubilized in 1X of IAP buffer (Cell Signaling). Samples were spinned at 13000 rpm for 3 min and cooled on ice. Wash the AC-Lysine agarose beads (Cell Signaling) 4 times with 1 ml of PBS. Add peptides to the Eppendorf tubes containing the Ac-Lys agarose beads. Samples were incubated for 2 hours at 4°C under constant rotation. Samples were spinned at 2000 g for 30 s, and supernatants were

discarded. Beads were washed 2 times with cold IAP buffer (1 ml) and 3 times with cold 20 mM Tris-HCl pH 7.6. Peptides were eluted from the beads with 100 ml of 0.15% TFA and desalted on stage tips. Samples were resolubilized in 4% formic acid (FA). Peptides were loaded and separated on a home-made reversed-phase column (150-µm i.d. by 200 mm) with a 106-min gradient from 10 to 30% ACN-0.2% FA and a 600-nl/min flow rate on an Easy nLC-1000 connected to an Orbitrap Fusion (Thermo Fisher Scientific, San Jose, CA). Each full MS spectrum acquired at a resolution of 120,000 was followed by tandem-MS (MS-MS) spectra acquisition on the most abundant multiply charged precursor ions for a maximum of 3s. Tandem-MS experiments were performed using collision-induced dissociation (CID) at a collision energy of 30%. The data were processed using PEAKS X Pro (Bioinformatics Solutions, Waterloo, ON) and a Uniprot human database (20366 entries). Mass tolerances on precursor and fragment ions were 10 ppm and 0.3 Da, respectively. The fixed modification was carbamidomethyl (C). Variable selected posttranslational modifications were oxidation (M), deamidation (NQ), phosphorylation (STY), and acetylation (N-ter). The data were visualized with Scaffold 4.0 (protein threshold, 99%, with at least 2 peptides identified and a false-discovery rate [FDR] of 1% for peptides).

Supplementary Figures and Supplementary Figure legends

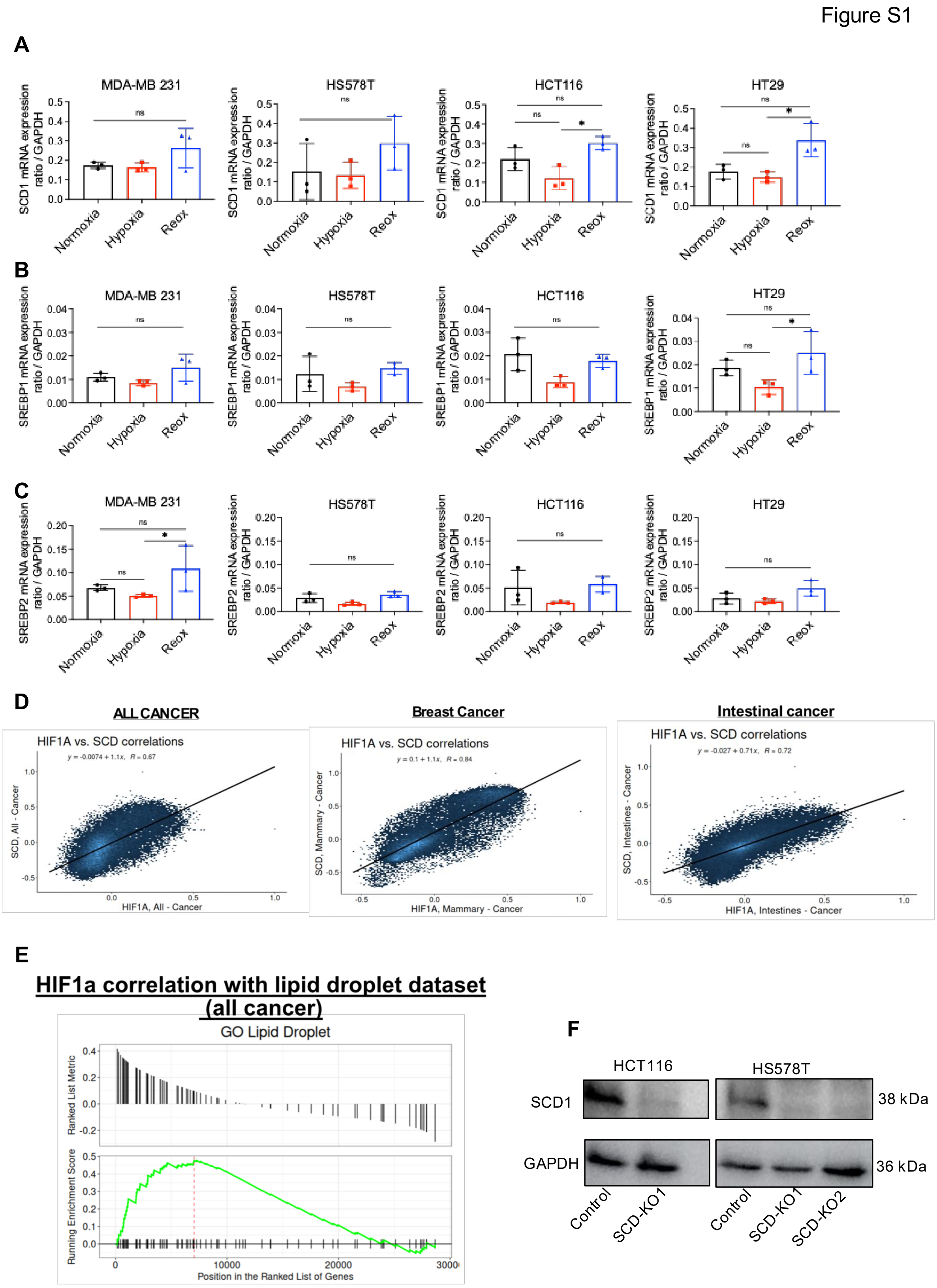

Figure S1: SCD1 expression is regulated by hypoxia and correlates with HIF1α in human cancers. (A–C) qRT-PCR analysis of SCD1 (A), SREBP1 (B), and SREBP2 (C) in MDA-MB231, HS578T, HCT116 and HT29 cells incubated under normoxic or hypoxic conditions or after 24 h reoxygenation (Reox) (n=3). Expression was normalized to GAPDH. (D) Scatter plots showing genome-wide co-expression of HIF1α and SCD1 across all cancers (left), breast cancers (middle), and intestinal cancers (right). (E) Gene Ontology (GO) analysis of ARCHS4 RNA-seq data identifies a lipid droplet signature significantly correlated with HIF1α. Genes were ranked by correlation with HIF1α using Correlation Analyzer, which calculated normalized enrichment scores (NES) and q-values. R values represent Pearson correlations. ARCHS4 v8 (Feb 2020) includes 238,522 human samples (Illumina HiSeq/NextSeq) linked to GEO entries with tissue annotations. (F) Western blot analysis of SCD1 expression in control and in SCD-KO HCT116 and HS578T cells. HSC70 served as a loading control. * $P < 0.05$

Figure S2

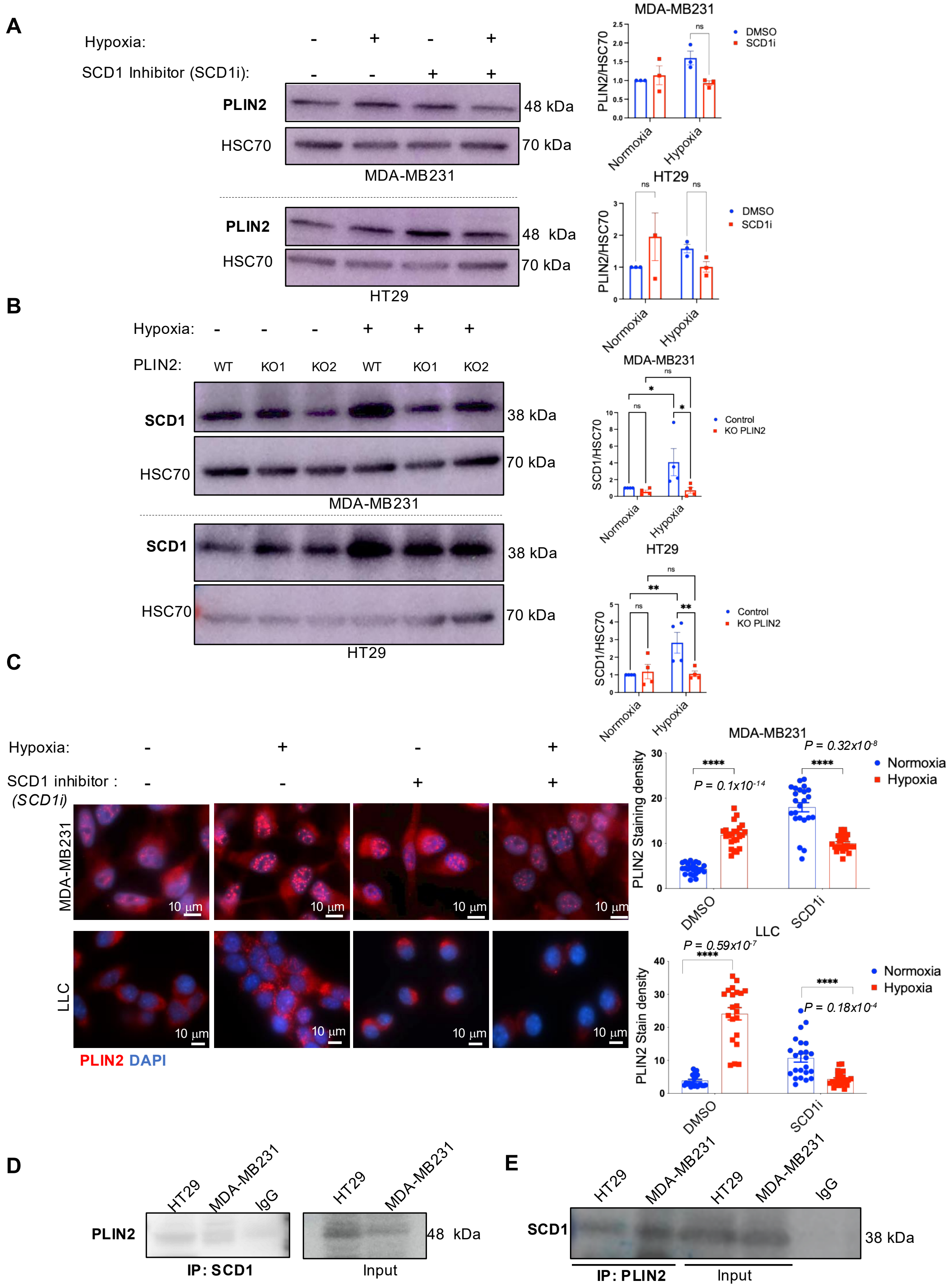
A
Hypoxia: - + - +
SCD1 Inhibitor (SCD1i): - - + +
PLIN2
48 kDa
HSC70
70 kDa
MDA-MB231
PLIN2
48 kDa
HSC70
70 kDa
HT29
MDA-MB231
PLIN2/HSC70
ns
DMSO
SCD1i
Normoxia
Hypoxia
HT29
ns
ns
B
Hypoxia: - - - + + +
PLIN2: WT KO1 KO2 WT KO1 KO2
SCD1
38 kDa
HSC70
70 kDa
MDA-MB231
SCD1
38 kDa
HSC70
70 kDa
HT29
SCD1/HSC70
Control
KO PLIN2
C
Hypoxia: - + - +
SCD1 inhibitor : - - + +
(SCD1i)
MDA-MB231
LLC
10 µm
PLIN2 DAPI
MDA-MB231
P = 0.32x10-8
P = 0.1x10-14
****
PLIN2 Staining density
Normoxia
Hypoxia
DMSO
SCD1i
LLC
P = 0.59x10-7
P = 0.18x10-4
PLIN2 Stain density
D
HT29
MDA-MB231
IgG
PLIN2
48 kDa
IP: SCD1
Input
E
SCD1
38 kDa
IP: PLIN2
Input

Figure S2: SCD1 and PLIN2 are mutually influenced and regulate LD formation in cancer cells. (A) Western blot of PLIN2 in MDA-MB231 and HT29 cells incubated under normoxic or hypoxic conditions and treated with DMSO (-) or SCD1 inhibitor (SCD1i; 20 μM) for 24 h. HSC70 served as a loading control and the graphs show the results of the densitometry quantification of the Western blot results. (B) Western blot of SCD1 on PLIN2 depleted from MDA-MB231 and HT29 Knockout (KO) cells incubated under normoxia or hypoxia conditions and quantifications. HSC70 served as a loading control. (C) PLIN2 staining on MDA-MB231 and LLC cells incubated either under normoxia or hypoxia conditions, with or without an SCD1 pharmaceutical inhibitor (SCD1i; 20 μM) and quantifications. (D) Detection of PLIN2 by Western blot analysis after immunoprecipitation of SCD1 in HT29 and MDA-MB231 cells lysates. (E) Reciprocal immunoprecipitation showing the detection of SCD1 by Western blot following immunoprecipitation of PLIN2 in HT29 and MDA-MB-231 cell lysates. Experiments were independently repeated three times (n=3). **P < 0.05; **P < 0.01; ****P < 0.001.*

Figure S3

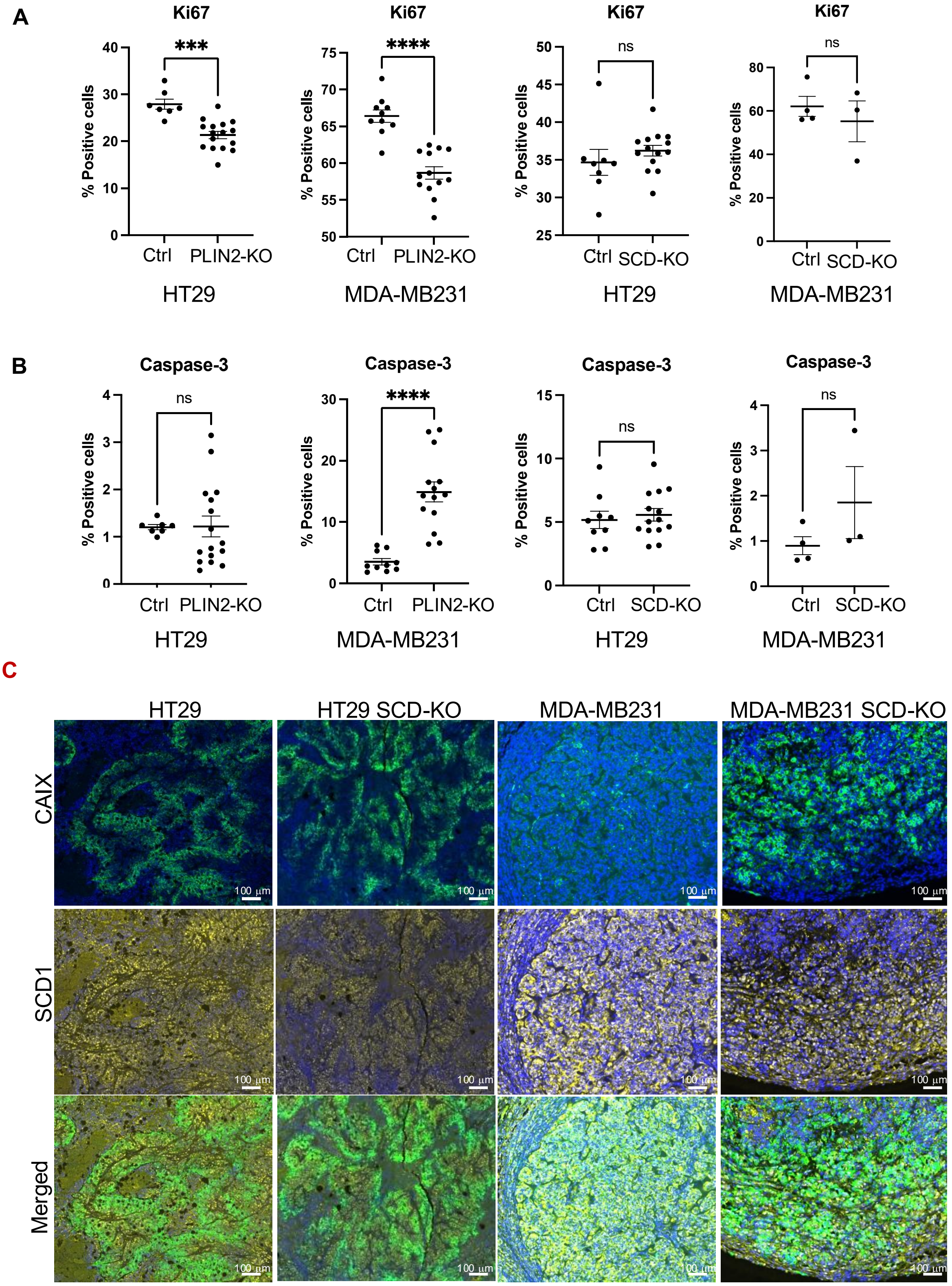

Figure S3: Depletion of PLIN2 decreases cell proliferation and increases apoptosis in vivo. (A, B) Immunohistochemistry analysis of Ki67 in (A) and caspase-3 in (B) in tumor

sections of HT29 and MDA-MB-231 xenografts depleted from PLIN2 or SCD1 and quantification of Ki67 or casapse-3 positive cells in whole tumor sections. (C) Immunofluorescent staining of CAIX (green), SCD1 (yellow), and DAPI (blue) in HT29 and MDA-MB231 control tumors, as well as in SCD-KO tumors, showing the whole section and the adjacent localization of CAIX and SCD1. Scale bars = 100 mm. *ns: P> 0.05; ***P< 0.001; **** P<0.0001*

Figure S4

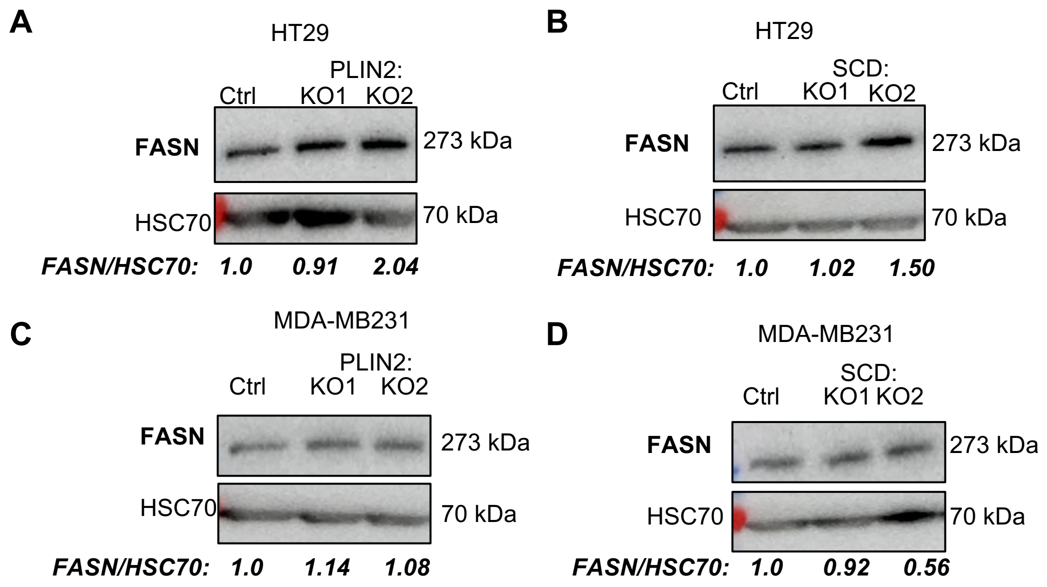

Figure S4: Inhibition of PLIN2 or SCD1 affects lipid synthesis in cancer cells. (A, B) Western blot analysis of FASN expression in control vs PLIN2 knockout (PLIN2-KO) (A) and in control vs. SCD1 knockout (SCD-KO) HT29 cells (B). (C, D) Western blot of FASN expression in control vs. PLIN2-KO (C) and in control vs. SCD-KO MDA-MB 231 cells (D). HSC70 served as a loading control. FASN densitometry analysis ratios were normalized to HSC70, and the values are shown below the blots (n = 2).

Figure S5

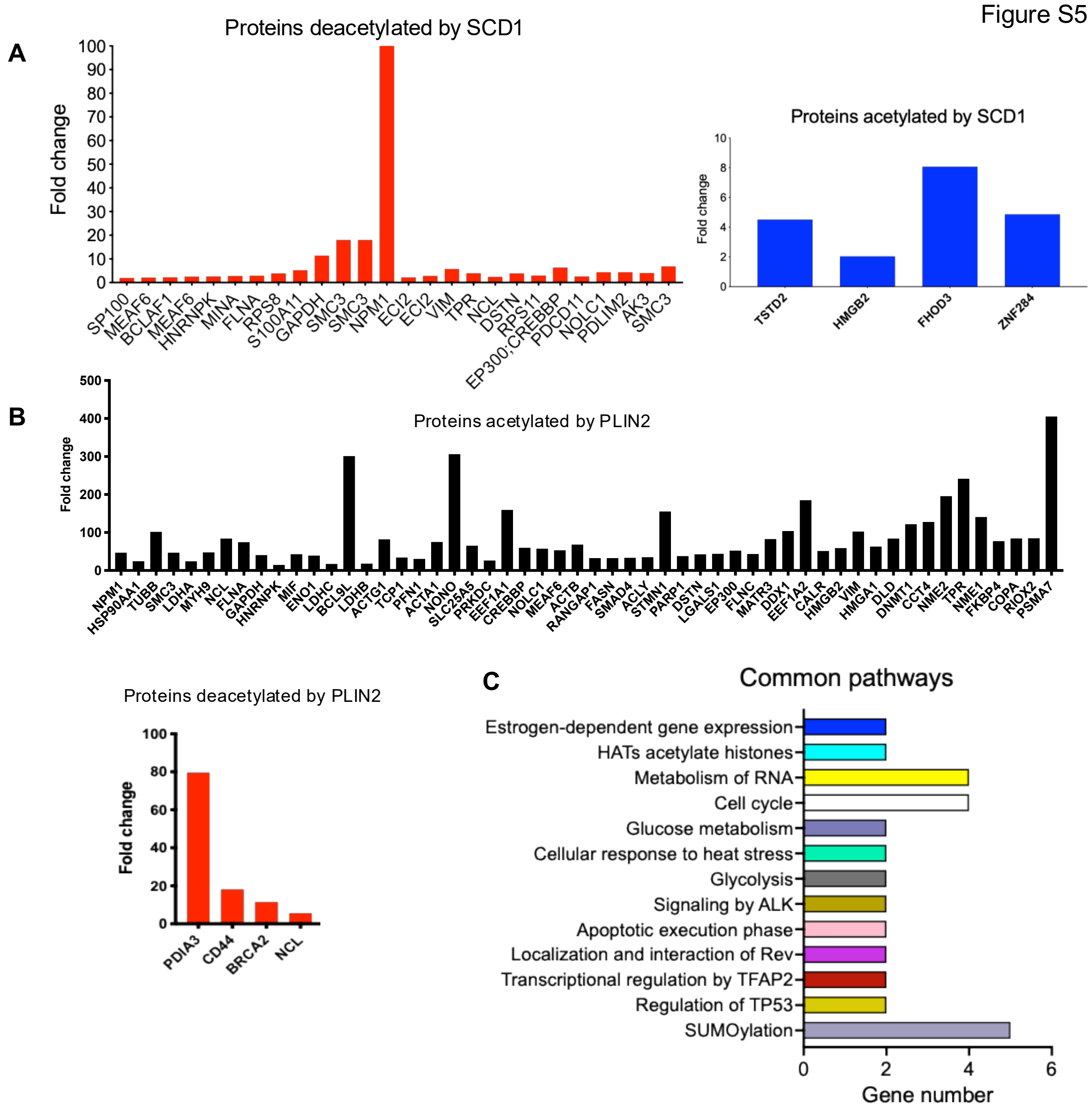

Figure S5: Downregulation of SCD1 increases the acetylation of non-histone proteins. (A) Acetylome analysis of nonhistone proteins deacetylated (left graph) or acetylated (right graph) in an SCD1-dependent manner in control MDA-MB231 cells compared to SCD1 depleted (SCD1-KD) cells. (B) Acetylome analysis of nonhistone proteins acetylated (top graph) or deacetylated (bottom graph) in an PLIN2-dependent manner in control MDA-MB231 cells compared to PLIN2 depleted (PLIN2-KO) cells (non-exhaustive). (C) Pathway enrichment analysis of proteins identified in both acetylome analyses.

Figure S6

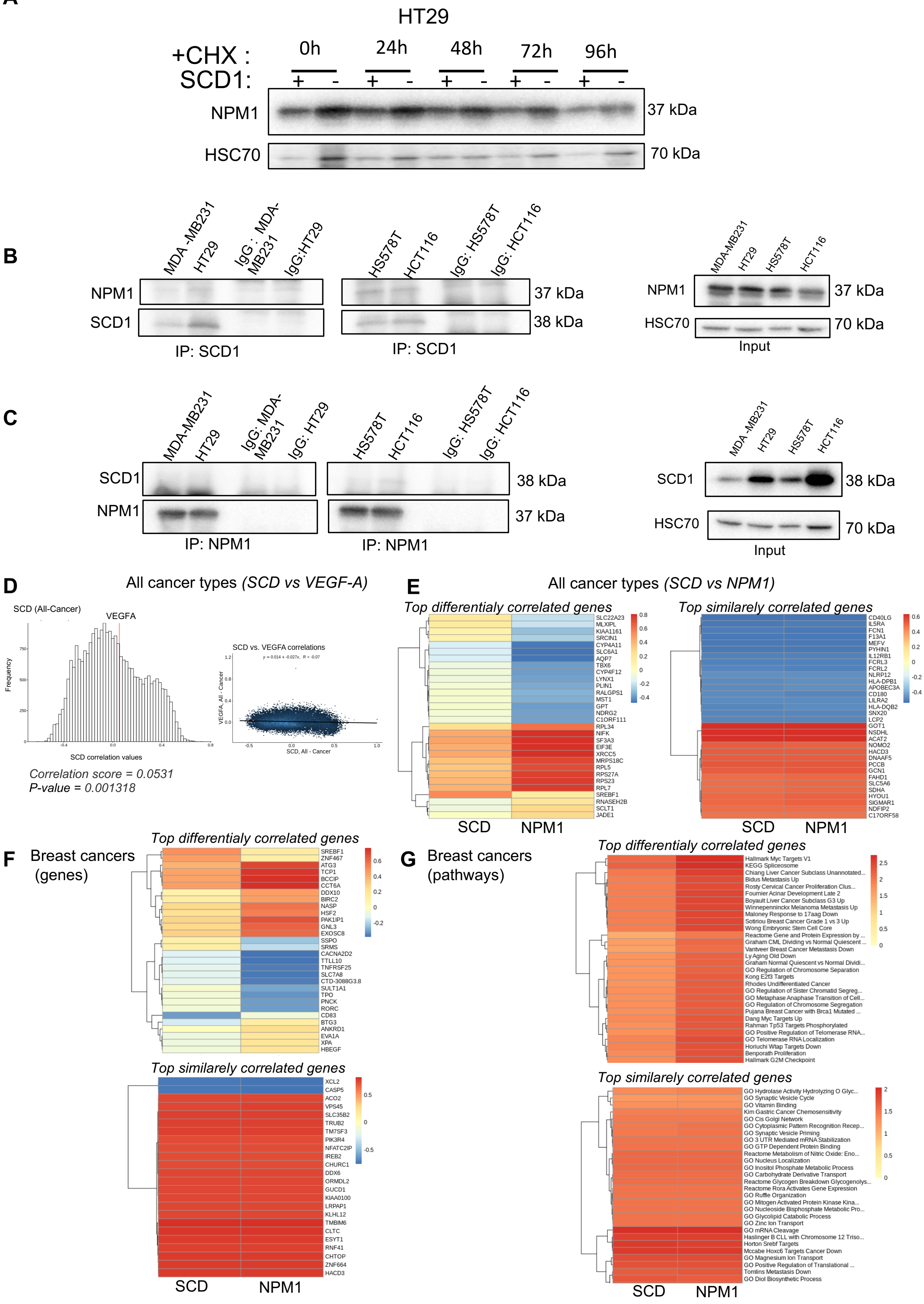
A
HT29
+CHX :
SCD1:
0h
24h
48h
72h
96h
+ - + - + - + - + -
NPM1
37 kDa
HSC70
70 kDa
B
MDA -MB231
HT29
IgG : MDA-MB231
IgG:HT29
HS578T
HCT116
IgG: HS578T
IgG: HCT116
NPM1
SCD1
37 kDa
38 kDa
IP: SCD1
IP: SCD1
MDA-MB231
HT29
HS578T
HCT116
NPM1
37 kDa
HSC70
70 kDa
Input
C
MDA-MB231
HT29
IgG: MDA-MB231
IgG: HT29
HS578T
HCT116
IgG: HS578T
IgG: HCT116
SCD1
NPM1
38 kDa
37 kDa
IP: NPM1
IP: NPM1
MDA -MB231
HT29
HS578T
HCT116
SCD1
38 kDa
HSC70
70 kDa
Input
D
All cancer types (SCD vs VEGF-A)
SCD (All-Cancer)
VEGFA
Frequency
SCD correlation values
SCD vs. VEGFA correlations
VEGFA, All - Cancer
SCD, All - Cancer
Correlation score = 0.0531
P-value = 0.001318
E
All cancer types (SCD vs NPM1)
Top differentialy correlated genes
SLC22A23
MLXIPL
KIAA1161
SRCIN1
CYP4A11
SLC6A1
AQP7
TBX6
CYP4F12
LYNX1
PLIN1
RALGPS1
MST1
GPT
NDRG2
C1ORF111
RPL34
NIFK
SF3A3
EIF3E
XRCC5
MRPS18C
RPL5
RPS27A
RPS23
RPL7
SREBF1
RNASEH2B
SCLT1
JADE1
SCD
NPM1
Top similarely correlated genes
CD40LG
IL5RA
FCN1
F13A1
MEFV
PYHIN1
IL12RB1
FCRL3
FCRL2
NLRP12
HLA-DPB1
APOBEC3A
CD180
LILRA2
HLA-DQB2
SNX20
LCP2
GOT1
NSDHL
ACAT2
NOMO2
HACD3
DNAAF5
PCCB
GCN1
FAHD1
SLC5A6
SDHA
HYOU1
SIGMAR1
NDFIP2
C17ORF58
SCD
NPM1
F
Breast cancers (genes)
Top differentialy correlated genes
SREBF1
ZNF467
ATG3
TCP1
BCCIP
CCT6A
DDX10
BIRC2
NASP
HSF2
PAK1IP1
GNL3
EXOSC8
SSPO
SRMS
CACNA2D2
TTLL10
TNFRSF25
SLC7A8
CTD-3088G3.8
SULT1A1
TPO
PNCK
RORC
CD83
BTG3
ANKRD1
EVA1A
XPA
HBEGF
Top similarely correlated genes
XCL2
CASP5
ACO2
VPS45
SLC35B2
TRUB2
TM7SF3
PIK3R4
NFATC2IP
IREB2
CHURC1
DDX6
ORMDL2
GUCD1
KIAA0100
LRPAP1
KLHL12
TMBIM6
CLTC
ESYT1
RNF41
CHTOP
ZNF664
HACD3
SCD
NPM1
G
Breast cancers (pathways)
Top differentialy correlated genes
Hallmark Myc Targets V1
KEGG Spliceosome
Chiang Liver Cancer Subclass Unannotated...
Bidus Metastasis Up
Rosty Cervical Cancer Proliferation Clus...
Fournier Acinar Development Late 2
Boyault Liver Cancer Subclass G3 Up
Winnepenninckx Melanoma Metastasis Up
Maloney Response to 17aag Down
Sotiriou Breast Cancer Grade 1 vs 3 Up
Wong Embryonic Stem Cell Core
Reactome Gene and Protein Expression by ...
Graham CML Dividing vs Normal Quiescent ...
Vantveer Breast Cancer Metastasis Down
Ly Aging Old Down
Graham Normal Quiescent vs Normal Dividi...
GO Regulation of Chromosome Separation
Kong E2f3 Targets
Rhodes Undifferentiated Cancer
GO Regulation of Sister Chromatid Segreg...
GO Metaphase Anaphase Transition of Cell...
GO Regulation of Chromosome Segregation
Pujana Breast Cancer with Brca1 Mutated ...
Dang Myc Targets Up
Rahman Tp53 Targets Phosphorylated
GO Positive Regulation of Telomerase RNA...
GO Telomerase RNA Localization
Horiuchi Wtap Targets Down
Benporath Proliferation
Hallmark G2M Checkpoint
Top similarely correlated genes
GO Hydrolase Activity Hydrolyzing O Glyc...
GO Synaptic Vesicle Cycle
GO Vitamin Binding
Kim Gastric Cancer Chemosensitivity
GO Cis Golgi Network
GO Cytoplasmic Pattern Recognition Recep...
GO Synaptic Vesicle Priming
GO 3 UTR Mediated mRNA Stabilization
GO GTP Dependent Protein Binding
Reactome Metabolism of Nitric Oxide: Eno...
GO Nucleus Localization
GO Inositol Phosphate Metabolic Process
GO Carbohydrate Derivative Transport
Reactome Glycogen Breakdown Glycogenolys...
Reactome Rora Activates Gene Expression
GO Ruffle Organization
GO Mitogen Activated Protein Kinase Kina...
GO Nucleoside Bisphosphate Metabolic Pro...
GO Glycolipid Catabolic Process
GO Zinc Ion Transport
GO mRNA Cleavage
Haslinger B CLL with Chromosome 12 Triso...
Horton Srebf Targets
Mccabe Hoxc6 Targets Cancer Down
GO Magnesium Ion Transport
GO Positive Regulation of Translational ...
Tomlins Metastasis Down
GO Diol Biosynthetic Process
SCD
NPM1

Figure S6: SCD interacts with NMP1 and increases its stabilization.

(A) Stability assay for NPM1 by Western blot at different time points after treatment with cycloheximide (CHX) (100 µM) in HT29 control and SCD1 knockout (SCD-KO) HT29 cells. (B) Immunoprecipitation of SCD1 and detection of NPM1 by Western blot analysis in MDA-MB231, HS578T, HCT116 and HT29 cells. Inputs are shown on the right. (C) Revers IP, after immunoprecipitation of NPM1 and detection of SCD1 by Western blot analysis. Inputs are shown on the right. HSC70 served as a loading control. (D) Correlation analysis of *SCD* gene expression with *VEGFA* gene (left graph) and Scatter plot (right graph) illustrating no correlation with these two genes in all type of cancers, correlation score of *0,0531* and *P-value = 0,001318*. (E) Top differentially (left) and similarly (right) correlated target genes between SCD and NPM1 in all cancer types. (F, G) Top differentially (left) and similarly (right) correlated target genes (F) and pathways (G) between SCD and NPM1 in breast cancers. Displayed R value determined by Pearson correlation.

Figure S7

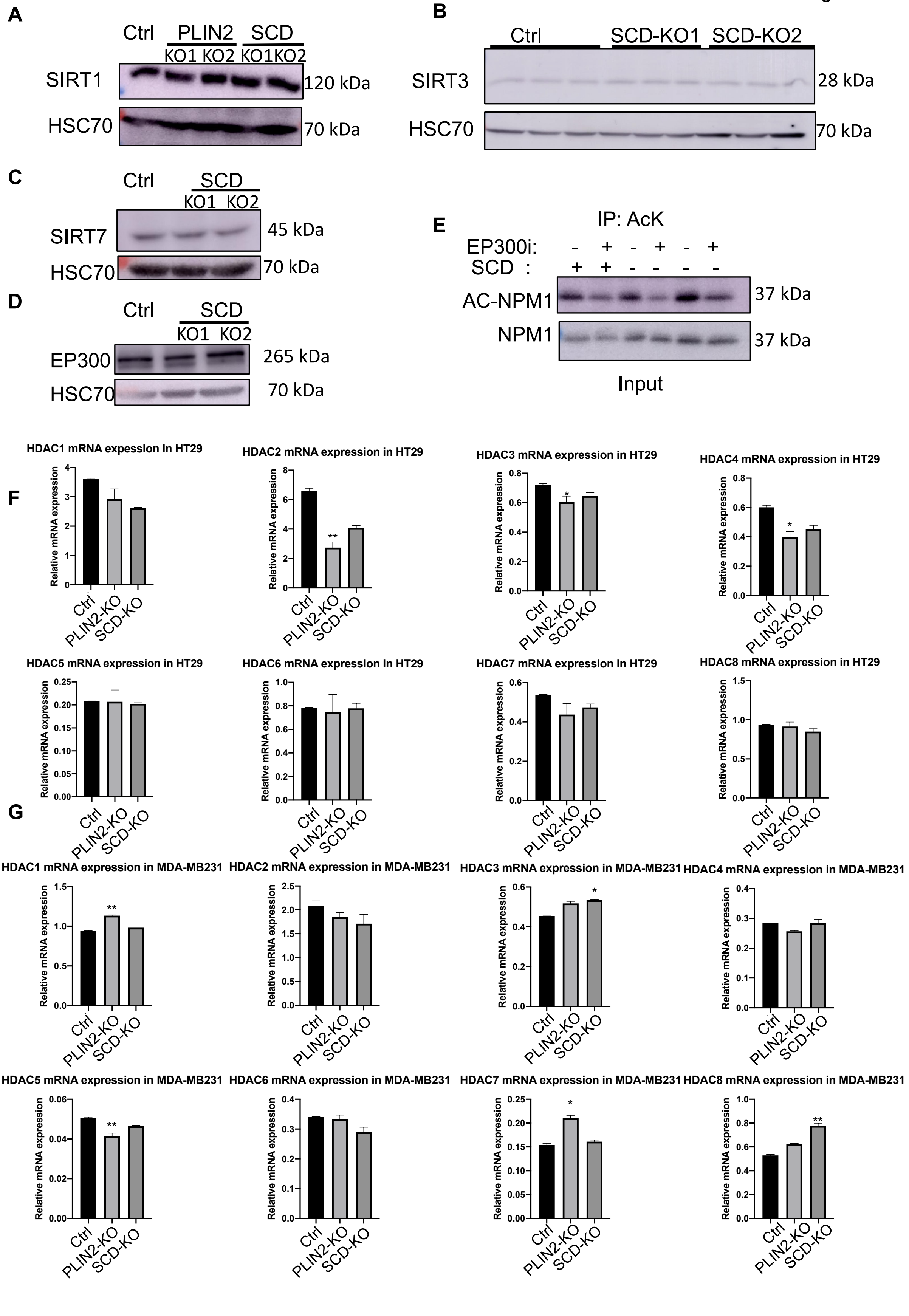
A
Ctrl
PLIN2
SCD
KO1 KO2 KO1 KO2
SIRT1
120 kDa
HSC70
70 kDa
B
Ctrl
SCD-KO1
SCD-KO2
SIRT3
28 kDa
HSC70
70 kDa
C
Ctrl
SCD
KO1 KO2
SIRT7
45 kDa
HSC70
70 kDa
D
Ctrl
SCD
KO1 KO2
EP300
265 kDa
HSC70
70 kDa
E
IP: AcK
EP300i: - + - + - +
SCD : + + - - - -
AC-NPM1
37 kDa
NPM1
37 kDa
Input
F
HDAC1 mRNA expression in HT29
HDAC2 mRNA expression in HT29
HDAC3 mRNA expression in HT29
HDAC4 mRNA expression in HT29
HDAC5 mRNA expression in HT29
HDAC6 mRNA expression in HT29
HDAC7 mRNA expression in HT29
HDAC8 mRNA expression in HT29
G
HDAC1 mRNA expression in MDA-MB231
HDAC2 mRNA expression in MDA-MB231
HDAC3 mRNA expression in MDA-MB231
HDAC4 mRNA expression in MDA-MB231
HDAC5 mRNA expression in MDA-MB231
HDAC6 mRNA expression in MDA-MB231
HDAC7 mRNA expression in MDA-MB231
HDAC8 mRNA expression in MDA-MB231
Relative mRNA expression
Ctrl
PLIN2-KO
SCD-KO

Figure S7: Effects of SCD1 and PLIN2 depletion on the expression of HDACs. (A–D) Western blot analysis of the deacetylases SIRT1 (A), SIRT3 (B), and SIRT7 (C), and the acetyltransferase EP300 (D) in control (Ctrl), PLIN2-KO or SCD-KO MDA-MB-231 cells. HSC70 served as a loading control. (E) Assessment of NPM1 acetylation by immunoprecipitation with an acetyl-lysine antibody (Ac-K), followed by western blotting for NPM1 in control (Ctrl) and SCD-KO cells treated with DMSO or the EP300 inhibitor (EP300i; 50 μM) for 24h. (F-G) qRT-PCR analysis of several mRNAs of HDACs in control, PLIN2-KO or SCD-KO HT29 cancer cells (F), and in control, PLIN2-KO or SCD1-KO MDA-MB231 cells (G).

Figure S8

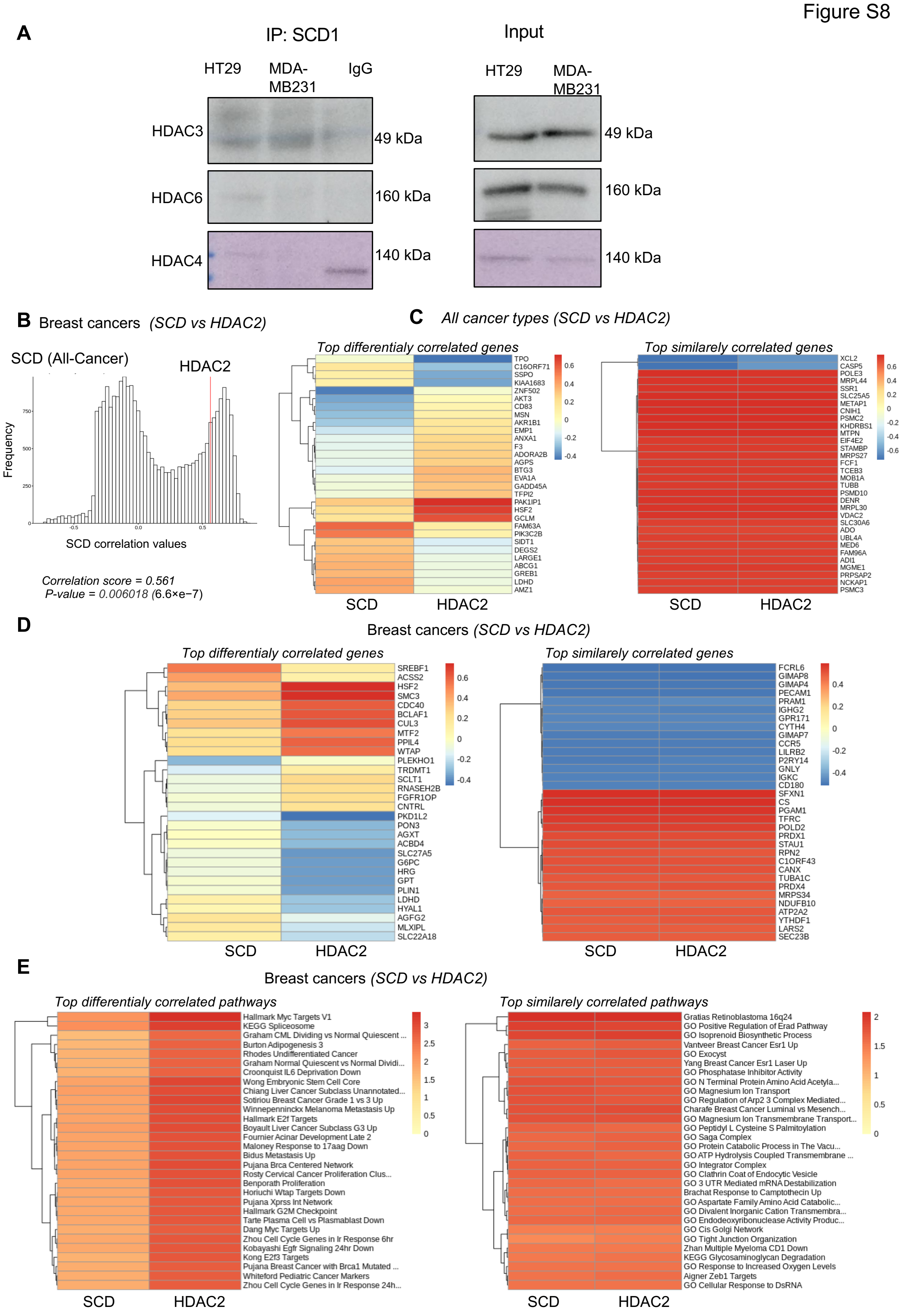

A
IP: SCD1
Input
HT29
MDA-MB231
IgG
HDAC3
HDAC6
HDAC4
49 kDa
160 kDa
140 kDa
B Breast cancers (SCD vs HDAC2)
SCD (All-Cancer)
HDAC2
Frequency
SCD correlation values
Correlation score = 0.561
P-value = 0.006018 (6.6×e−7)
C All cancer types (SCD vs HDAC2)
Top differentialy correlated genes
TPO
C16ORF71
SSPO
KIAA1683
ZNF502
AKT3
CD83
MSN
AKR1B1
EMP1
ANXA1
F3
ADORA2B
AGPS
BTG3
EVA1A
GADD45A
TFPI2
PAK1IP1
HSF2
GCLM
FAM63A
PIK3C2B
SIDT1
DEGS2
LARGE1
ABCG1
GREB1
LDHD
AMZ1
Top similarely correlated genes
XCL2
CASP5
POLE3
MRPL44
SSR1
SLC25A5
METAP1
CNIH1
PSMC2
KHDRBS1
MTPN
EIF4E2
STAMBP
MRPS27
FCF1
TCEB3
MOB1A
TUBB
PSMD10
DENR
MRPL30
VDAC2
SLC30A6
ADO
UBL4A
MED6
FAM96A
ADI1
MGME1
PRPSAP2
NCKAP1
PSMC3
SCD
HDAC2
D
Breast cancers (SCD vs HDAC2)
Top differentialy correlated genes
SREBF1
ACSS2
HSF2
SMC3
CDC40
BCLAF1
CUL3
MTF2
PPIL4
WTAP
PLEKHO1
TRDMT1
SCLT1
RNASEH2B
FGFR1OP
CNTRL
PKD1L2
PON3
AGXT
ACBD4
SLC27A5
G6PC
HRG
GPT
PLIN1
LDHD
HYAL1
AGFG2
MLXIPL
SLC22A18
Top similarely correlated genes
FCRL6
GIMAP8
GIMAP4
PECAM1
PRAM1
IGHG2
GPR171
CYTH4
GIMAP7
CCR5
LILRB2
P2RY14
GNLY
IGKC
CD180
SFXN1
CS
PGAM1
TFRC
POLD2
PRDX1
STAU1
RPN2
C1ORF43
CANX
TUBA1C
PRDX4
MRPS34
NDUFB10
ATP2A2
YTHDF1
LARS2
SEC23B
SCD
HDAC2
E
Breast cancers (SCD vs HDAC2)
Top differentialy correlated pathways
Hallmark Myc Targets V1
KEGG Spliceosome
Graham CML Dividing vs Normal Quiescent ...
Burton Adipogenesis 3
Rhodes Undifferentiated Cancer
Graham Normal Quiescent vs Normal Dividi...
Croonquist IL6 Deprivation Down
Wong Embryonic Stem Cell Core
Chiang Liver Cancer Subclass Unannotated...
Sotiriou Breast Cancer Grade 1 vs 3 Up
Winnepenninckx Melanoma Metastasis Up
Hallmark E2f Targets
Boyault Liver Cancer Subclass G3 Up
Fournier Acinar Development Late 2
Maloney Response to 17aag Down
Bidus Metastasis Up
Pujana Brca Centered Network
Rosty Cervical Cancer Proliferation Clus...
Benporath Proliferation
Horiuchi Wtap Targets Down
Pujana Xprss Int Network
Hallmark G2M Checkpoint
Tarte Plasma Cell vs Plasmablast Down
Dang Myc Targets Up
Zhou Cell Cycle Genes in Ir Response 6hr
Kobayashi Egfr Signaling 24hr Down
Kong E2f3 Targets
Pujana Breast Cancer with Brca1 Mutated ...
Whiteford Pediatric Cancer Markers
Zhou Cell Cycle Genes in Ir Response 24h...
Top similarely correlated pathways
Gratias Retinoblastoma 16q24
GO Positive Regulation of Erad Pathway
GO Isoprenoid Biosynthetic Process
Vantveer Breast Cancer Esr1 Up
GO Exocyst
Yang Breast Cancer Esr1 Laser Up
GO Phosphatase Inhibitor Activity
GO N Terminal Protein Amino Acid Acetyla...
GO Magnesium Ion Transport
GO Regulation of Arp2 3 Complex Mediated...
Charafe Breast Cancer Luminal vs Mesench...
GO Magnesium Ion Transmembrane Transport...
GO Peptidyl L Cysteine S Palmitoylation
GO Saga Complex
GO Protein Catabolic Process in The Vacu...
GO ATP Hydrolysis Coupled Transmembrane ...
GO Integrator Complex
GO Clathrin Coat of Endocytic Vesicle
GO 3 UTR Mediated mRNA Destabilization
Brachat Response to Camptothecin Up
GO Aspartate Family Amino Acid Catabolic...
GO Divalent Inorganic Cation Transmembra...
GO Endodeoxyribonuclease Activity Produc...
GO Cis Golgi Network
GO Tight Junction Organization
Zhan Multiple Myeloma CD1 Down
KEGG Glycosaminoglycan Degradation
GO Response to Increased Oxygen Levels
Aigner Zeb1 Targets
GO Cellular Response to DsRNA
SCD
HDAC2

Figure S8: SCD1 and HDAC2 gene expression correlated in human cancer. (A) Co-immunoprecipitation of SCD1 and western blot analysis of HDAC3, HDAC4 and HDAC6 in HT29 and MDA-MB231 cells. Inputs are shown on the right. (B) Correlation score of *SCD* and *HDAC2* genes in breast cancers, correlation score of *0.561 and P-value = 0.006*. (C) Top differentially (left) and similarly (right) correlated target genes between SCD and HDAC2 in all cancer types. (D, E) Top differentially (left) and similarly (right) correlated target genes (E) and pathways (E) between *SCD* and *HDAC2* genes in breast cancers. Displayed R value determined by Pearson correlation.

Figure S9

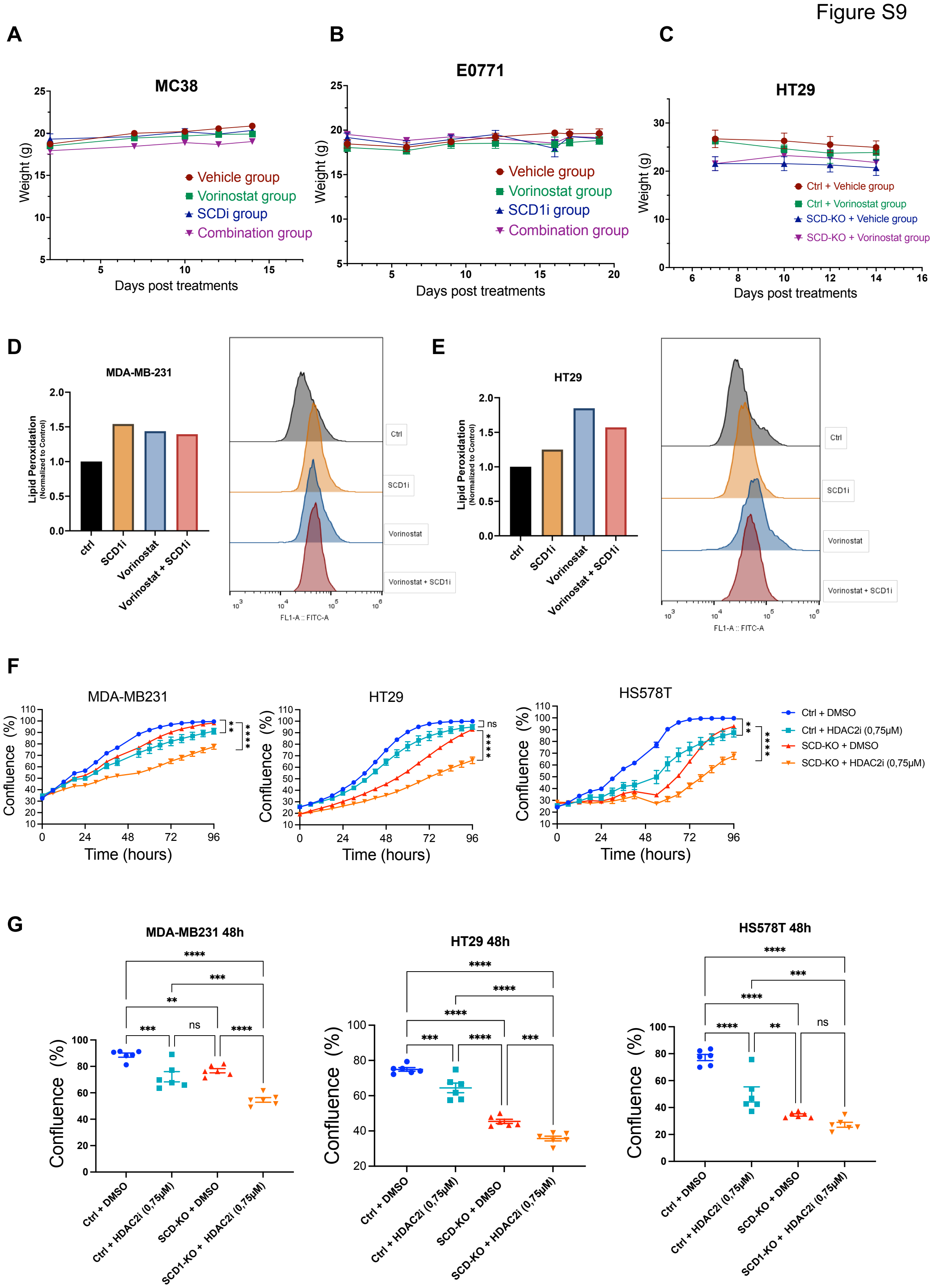
A
MC38
Weight (g)
Days post treatments
Vehicle group
Vorinostat group
SCDi group
Combination group
B
E0771
Weight (g)
Days post treatments
Vehicle group
Vorinostat group
SCD1i group
Combination group
C
HT29
Weight (g)
Days post treatments
Ctrl + Vehicle group
Ctrl + Vorinostat group
SCD-KO + Vehicle group
SCD-KO + Vorinostat group
D
MDA-MB-231
Lipid Peroxidation
(Normalized to Control)
ctrl
SCD1i
Vorinostat
Vorinostat + SCD1i
Ctrl
SCD1i
Vorinostat
Vorinostat + SCD1i
FL1-A :: FITC-A
E
HT29
Lipid Peroxidation
(Normalized to Control)
ctrl
SCD1i
Vorinostat
Vorinostat + SCD1i
Ctrl
SCD1i
Vorinostat
Vorinostat + SCD1i
FL1-A :: FITC-A
F
MDA-MB231
HT29
HS578T
Confluence (%)
Time (hours)
ns
Ctrl + DMSO
Ctrl + HDAC2i (0,75µM)
SCD-KO + DMSO
SCD-KO + HDAC2i (0,75µM)
G
MDA-MB231 48h
HT29 48h
HS578T 48h
Confluence (%)
ns
Ctrl + DMSO
Ctrl + HDAC2i (0,75µM)
SCD-KO + DMSO
SCD1-KO + HDAC2i (0,75µM)
SCD-KO + HDAC2i (0,75µM)

Figure S9: SCD-KO cells and tumors show sensitivity to acetylation inhibitors. (A) Weight of C57BL/6 mice bearing MC38 tumors treated with vehicle, vorinostat, an SCD inhibitor (SCDi), or a combination of vorinostat and SCDi. (B) Weight of C57BL/6 mice bearing E0771 tumors treated with vehicle, vorinostat, SCDi, or the combination of vorinostat and SCDi. (C) Weight of RAG1-/- mice bearing HT29 control or SCD-KO tumors treated with vehicle or vorinostat. (D–E) Lipid peroxide detection using BODIPY™ 581/591 C11 by FACS in (D) MDA-MB-231 cells or (E) HT29 cells treated with DMSO, SCD1 inhibitor (SCD1i; 20 μM), vorinostat (0.75 μM), or both inhibitors for 24 h. Fold change of mean fluorescence intensity (MFI) relative to the control group (Ctrl) was calculated for each condition; shift counts are shown on the right. (F) Proliferation assay with the *Incucyte*® at the indicated time points for MDA-MB-231, HT29, and HS578T control (Ctrl) cells and their corresponding SCD-KO cells treated with DMSO or the HDAC2 inhibitor santacruzamate A (0.75 μM). (G) Cell proliferation at 48 h of the cell lines described in panel (F). The FACS experiments were independently reproduced twice, and the *Incucyte* assays were reproduced three times.
